# Home Health Care and Hospice Use Among Medicare Beneficiaries With and Without a Diagnosis of Dementia

**DOI:** 10.1089/jpm.2023.0583

**Published:** 2024-06-22

**Authors:** Hyosin (Dawn) Kim, Paul R. Duberstein, Haiqun Lin, Bei Wu, Anum Zafar, Olga F. Jarrín

**Affiliations:** ^1^ College of Health, Oregon State University, Corvallis, Oregon, USA.; ^2^ School of Public Health, Rutgers, The State University of New Jersey, Piscataway, New Jersey, USA.; ^3^ School of Nursing, Rutgers, The State University of New Jersey, Newark, New Jersey, USA.; ^4^ Rory Meyers College of Nursing, New York, New York, USA.; ^5^ NYU Aging Incubator, New York University, New York, New York, USA.; ^6^ Institute for Health, Health Care Policy, and Aging Research, Rutgers, The State University of New Jersey, New Brunswick, New Jersey, USA.

**Keywords:** dementia, end-of-life, home health care, hospice, Medicare

## Abstract

**Background:**

Home health care is a core benefit of Medicare and Medicaid insurance programs and includes services to improve health, maintain health, or slow health decline.

**Objective:**

To examine the relationship between home health care use during the last three years of life and hospice use in the last six months of life among Medicare beneficiaries with and without dementia.

**Design:**

Nationally representative retrospective cohort study.

**Setting/Subjects:**

Medicare beneficiaries with at least three years of continuous enrollment who died in 2019 in the United States (*n* = 2,169,422).

**Measurements:**

The primary outcome was hospice use, and the secondary outcome was hospice duration. The independent variable was a composite of the presence and timing of home health care initiation during the last three years of life.

**Results:**

Home health care was used by 46.4% of Medicare beneficiaries and hospice care was used by 53.1% of beneficiaries, with 28.3% using both. Compared with beneficiaries who did not use home health care, those who started home health care before the last year of life (odds ratio [OR] = 1.57, 95% confidence interval [CI] = 1.56–1.58) or during the last year of life (OR = 1.75, 95% CI = 1.74–1.77) were more likely to use hospice. The effects were stronger in those without a diagnosis of dementia (OR = 1.92, 95% CI = 1.90–1.94) compared with those without a dementia diagnosis (OR = 1.34, 95% CI = 1.32–1.35) who started home health in the final year of life.

**Conclusions:**

Receiving home health care in the final years of life is associated with increased hospice use at the end-of-life in Medicare beneficiaries with and without a dementia diagnosis.

## Introduction

Over the last 10 years, there has been a notable shift in how Medicare addresses the needs of those with serious illnesses, moving from hospital settings to community-based care.^1,2^ The focus of end-of-life care has similarly shifted to more familiar, home-like environments.^3^ Home health care has become a cornerstone for supporting older adults with serious conditions, particularly dementia,^4^ which often requires complex care involving both informal caregivers and professional health providers.^5^ These caregivers not only offer medical assistance but also build a rapport with families, guiding them through end-of-life decisions with compassion and informed options. Data from the National Health and Aging Trends Study (NHATS) show that ∼39% of Medicare patients relied on home health services in their final years.^6^

Home health care encompasses a spectrum of services, including skilled nursing, therapy, social work, and aide services, when skilled care is needed to maintain function or to prevent or slow decline.^7^ Hospice care, provided either at home or in a facility, offers similar skilled services for those with life expectancies of six months or less, focusing on comfort and family support.^8^ Recipients of hospice care generally report higher satisfaction, feeling that their dignity and spiritual needs are honored.^9,10^ Despite this, research into the concurrent use of home health and hospice care is scant. One study of fee-for-service patients in the NHATS data (*n* = 1057) found that 19.9% of Medicare decedents used home health care alone, whereas 25.8% used hospice services, and 18.8% used both.^6^ Another study noted that 7%–9% of those in hospice had previously received home health care, with no significant change in this pattern from 2011 to 2018, but it did not extensively investigate the duration of care beyond the last 90 days of life.^11^

Some research has delved into how home-based care models, like those led by nurse practitioners or state-funded programs, affect hospice use. A Michigan study showed that patients receiving home-based care were more likely to be referred to hospice than those in nursing homes.^12^ Furthermore, a significant majority of dementia patients in nurse-led home care entered hospice, where nearly all had documented end-of-life care discussions.^13^ This reflects a model of care that emphasizes active patient and family engagement and is marked by lower hospital and emergency visits near death. Despite these findings, a national overview of the impact of home health services on hospice use, particularly for those with dementia, is still missing.

As the preference for aging at home grows,^14^ it's vital to understand how home health care affects the end-of-life experience, particularly for those with complex health issues.^2^ The benefits of hospice care in enhancing end-of-life quality for patients and caregivers are clear,^9,10,15^ prompting an investigation into how home health services may lead to increased hospice use. This study proposes that home health care's benefits last well past the care period, influencing future care decisions such as transitioning to hospice. It explores the link between home health care in the final three years of life and subsequent hospice use for individuals with and without dementia.

## Methods

### Design and participants

We conducted a retrospective cohort study of U.S. Medicare fee-for-service and Medicare Advantage beneficiaries who died in 2019 with a three-year look-back period. Our sample comprised 100% of Medicare beneficiaries who were aged 40 years or older at death, were continuously enrolled in Medicare for at least three years before death, and resided in 1 of the 50 states, the District of Columbia, Puerto Rico, or the U.S. Virgin Islands. At the beneficiary level, we linked sociodemographic and chronic conditions from the Master Beneficiary Summary File, and health care use from the inpatient Medicare Provider and Analysis Review (MedPAR) file, hospice claims, Minimum Data Set (MDS 3.0, MDS swing bed), Outcome and Assessment Information Set (OASIS), and inpatient rehabilitation facility-patient assessment instrument.

We also used the Medicare enrollment database for the 9-digit ZIP codes to link the data from the 2018 Area Deprivation Index 3.0 (ADI 3.0)^16^ and Rural Urban Continuum Codes (RUCC)^17^ to identify disadvantaged neighborhoods in urban or rural areas. The final beneficiary-level analytic dataset included a quarterly summary of inpatient and home hospice, home health care, nursing home, inpatient days and admissions for the last 3 years of life (12 quarters in total), and covariates described later (sociodemographic, geographic, and clinical variables).

### Measures

#### Hospice use and length of hospice care

The primary outcome was hospice use (either at home or inpatient) during the last six months of life. The secondary outcome was duration of hospice care: <3,^18–20^ 3–179, and ≥180 days.^21,22^ Hospice care lasting less than three days is often used as an indicator of poor end-of-life care quality and underuse of hospice and palliative care.^18–20^ Similarly, we follow the literature and report on hospice care lasting more than 180 days.^21,22^ Given that there is no consensus on quality indicators,^23^ for sensitivity analysis, a different cutoff for duration of hospice care was used for comparison of findings: ≤7, 8–179, or ≥180 days.

#### Use and timing of home health care

The main independent variable was a three-category indicator of home health use during the last three years of life: (1) no use of home health care, (2) home health care initiated before the last year of life, and (3) home health care initiated in the last year of life. This indicator is designed to assess whether earlier exposure to home health care increases hospice use. To calculate home health care days and assign them to each beneficiary, we used the dates of service from the OASIS assessment file for our sample during the study period from 2016 to 2019 (see Supplementary Data Text S1, Tables S5a and S5b). Our study expands the framework of end-of-life care from the last 12 months of life to the last 3 years of life.^24^ The look-back period of three years for our 2019 cohort also aligns with temporal changes in CMS data (change from ICD-9 to ICD-10 in 2015) and precedes the COVID-19 pandemic.

#### Covariates

Covariates included age at death, sex, insurance type (fee-for-service vs. Medicare Advantage, and dual eligibility for Medicaid), and race/ethnicity (administrative race data were augmented with self-reported race/ethnicity from postacute and long-term care assessment data).^25^ As Medicare is a national socialized health insurance program for people aged 65 years and older, and younger people with a disability, end-stage renal disease (ESRD), or amyotrophic lateral sclerosis or Lou Gehrig's disease,^26^ we included a binary variable (death before age 68) to distinguish Medicare eligibility based only on disability at the beginning of the look-back period. Chronic conditions flags were created using the MBSF chronic conditions segment (from first-ever occurrence dates) for ischemic heart disease, hypertension, hyperlipidemia, chronic kidney disease, depression, congestive heart failure, diabetes, chronic obstructive pulmonary disease, history of stroke/transient ischemic attack, cancer, acute myocardial infarction, and ESRD.

The warehouse indicators of chronic conditions for Alzheimer's disease and other dementias were augmented with other dementia diagnoses recorded in claims and assessment data (see Text S2 in Supplementary Data for details). The number of institutional care days was calculated using MedPAR summary claims for hospital admissions and days, MDS and MDS-Swing Bed data for skilled nursing facility admissions and days. Covariates for health care use included the number of acute hospitalizations and a binary indicator for ≥100 days in skilled nursing facilities. A neighborhood profile variable was created at the 9-digit zip code level by combining the ADI 3.0^16^ and rural–urban classification (RUCC)^17^ into four categories: urban-advantaged, urban-disadvantaged, rural-advantaged, and rural-disadvantaged.^27^ The patient's state of residence was also included as a fixed effect in the models to adjust for state variation.^21,28^

#### Analytic approach

First, we characterized the overall sample based on the use and timing of home health care. We then describe the sample characteristics and health care use among individuals with and without dementia. We reported the absolute counts and percentages of sociodemographic and clinical variables, including insurance type, neighborhood profile, chronic conditions, and health care utilization within the last three years of life. We reported quartiles of home health days, days in hospice care, acute hospital admissions, and days in skilled nursing facilities. We used chi-square tests for categorical variables and *t* tests for continuous variables to examine bivariate relationships.

Next, we conducted logistic regression analyses to assess the association between home health care use and the primary outcome of hospice use in the full sample and samples stratified by dementia diagnosis. In addition, we conducted logistic regression with the outcome of hospice use, including an interaction term between dementia diagnosis and home health use, and calculated predicted probabilities of hospice use by dementia and home health use. In the second main analysis, we performed a series of multinomial logistic regression analyses relating the relative probabilities for home health care use and categories of hospice duration with no hospice use as the reference category. As for hospice duration, we used two versions of the four categorical outcome measure (no hospice, 1–3 days, 3–179 days, ≥180 days; and no hospice, 1–7 days, 8–179 days, ≥180 days).

Logistic and multinomial regression models were adjusted for sociodemographic, geographic, and clinical factors. The adjusted analyses were also stratified based on dementia diagnosis. We performed two sensitivity analyses: one with a sample of decedents excluding those who received home health after being discharged from hospice (0.06% of our sample) and the other with a sample of decedents excluding those who died in a nursing home (*n* = 121,669, 5.6%).

## Results

### Sample description

Descriptive statistics for the overall sample stratified by use of home health care during the last three years of life are presented in Table 1. Of our sample of 2,169,422 Medicare decedents in 2019, 46.4% used home health in the last three years of life, with 28.8% starting home health care before the last year of life (*n* = 624,776), and 17.6% starting home health care during the last year of life (*n* = 380,905). These individuals who started home health care before the last year of life were more likely to have multiple chronic conditions (median = 7, interquartile range [IQR] = 5–9) compared with those who began using home health closer to death (median = 6, IQR = 4–9) and those who never used home health in the last three years of life (median = 5, IQR = 3–8).

**Table 1.** Characteristics of 2019 Medicare Decedents by Timing of Home Health Care Initiation During the Last Three Years of Life (Column %), *N* = 2,169,422

Variable	None	Last year	Before last year
Total sample, *n* (%)	1,163,741 (53.6)	380,905 (17.6)	624,776 (28.8)
Hospice use	539,036 (46.3)	231,564 (60.8)	381,366 (61.0)
Hospice days, median [IQR]	0 [0–13]	3 [0–18]	4 [0–36]
Age at death, mean (SD)	79.7 (11.0)	80.5 (10.0)	82.4 (10.4)
Age <68 at death	134,580 (11.6)	33,617 (8.8)	52,123 (8.3)
Female	574,015 (49.3)	191,650 (50.3)	357,712 (57.3)
Male	589,726 (50.7)	189,255 (49.7)	267,064 (42.8)
White, non-Hispanic	923,954 (79.4)	310,973 (81.6)	506,278 (81.0)
Black, non-Hispanic	113,566 (9.8)	37,980 (9.97)	67,194 (10.8)
Hispanic	90,184 (7.8)	21,955 (5.8)	36,491 (5.8)
Asian American/Pacific Islander	28,948 (2.5)	8,321 (2.2)	12,072 (1.9)
American Indian/Alaska Native	7089 (0.6)	1676 (0.4)	2741 (0.4)
Medicare FFS only	492,453 (42.3)	191,565 (50.3)	289,794 (46.4)
Medicare FFS-Medicaid dual	215,547 (18.5)	45,781 (12.0)	119,047 (19.1)
Medicare advantage only	316,613 (27.2)	110,226 (28.9)	144,814 (23.2)
Medicare advantage dual	139,128 (12.0)	33,333 (8.8)	71,131 (11.4)
Neighborhood profile			
Urban, advantaged zip code	776,738 (66.7)	264,040 (69.3)	437,238 (70.0)
Urban, disadvantaged zip code	166,035 (14.3)	48,014 (12.6)	72,171 (11.6)
Rural, advantaged zip code	113,118 (9.7)	35,224 (9.3)	60,272 (9.7)
Rural, disadvantaged zip code	107,850 (9.3)	33,627 (8.8)	55,095 (8.8)
Count of CCs, median [IQR]	5 [3–8]	6 [4–9]	7 [5–9]
Alzheimer's disease and related dementias	421,927 (36.3)	154,057 (40.4)	357,634 (57.2)
Ischemic heart disease	625,532 (53.8)	239,556 (62.9)	444,311 (71.1)
Hypertension	884,453 (76.0)	313,995 (82.4)	548,871 (87.9)
Hyperlipidemia	780,033 (67.0)	285,471 (75.0)	502,955 (80.5)
Chronic kidney disease	549,852 (47.3)	218,284 (57.3)	402,365 (64.4)
Depression	465,905 (40.0)	165,475 (43.4)	352,044 (56.4)
Congestive heart failure	481,654 (41.4)	196,384 (51.6)	385,642 (61.7)
Diabetes	460,591 (39.6)	174,372 (45.8)	332,316 (53.2)
COPD	408,958 (35.1)	162,230 (42.6)	313,146 (50.1)
Stroke/TIA	258,290 (22.2)	95,469 (25.1)	210,646 (33.7)
Cancer	230,270 (19.8)	98,407 (25.8)	146,275 (23.4)
Acute myocardial infarction	108,429 (9.3)	46,122 (12.1)	90,069 (14.4)
End-stage renal disease	30,438 (2.6)	16,596 (4.4)	33,156 (5.3)
Hospitalizations, median [IQR]^a^	1 [0–3]	3 [2–5]	4 [2–7]
SNF days, median [IQR]^a^	0 [0–29]	0 [0–27]	17 [0–65]
≥100 SNF days^a^	220,656 (19.0)	24,269 (6.4)	114,305 (18.3)

The sample descriptions based on a dementia diagnosis were also presented in Supplementary Tables S3 and S4.

Chi-square tests for categorical variables and analyses of variance for continuous variables were all statistically significant with a *p* < 0.001.

^a^ Health services utilization in the last three years was reported, except for hospice use, reported for the last six months of life.

CC, chronic conditions; COPD, chronic obstructive pulmonary disease; FFS, fee-for-service; IQR, interquartile range; SD, standard deviation; SNF, skilled nursing facility; TIA, transient ischemic attack.

Statistically significant racial/ethnic differences in the use of home health care services during the last three years of life were observed (*p* < 0.001). Compared with Black and White beneficiaries, home health care during the last three years of life was less likely to be used among Hispanic, Asian American/Pacific Islander, and American Indian/Alaska Native beneficiaries. See more information on racial/ethnic, rural/urban, and socioeconomic differences in Table 1 and Supplementary Data (Supplementary Table S1). Overall, 53.1% used hospice services in the last six months of life, ranging from 46.3% of those who did not use home health care to 61.0% of those who started home health before the last year of life. Of all the individuals using hospice during the last six months of life, 671 (0.06%) were admitted to home health care after being discharged from hospice, and later died while receiving home health care.

In our sample, 43.0% of Medicare beneficiaries were diagnosed with dementia during their lifetime (*n* = 933,618) (Table 2). Individuals with dementia were older (mean age, 84.5 years [standard deviation, SD = 9.4] vs. 77.7 years [SD = 10.7]) and presented with more chronic conditions (median of 8 chronic conditions compared with 5 for those without dementia). A greater proportion of dually enrolled beneficiaries had a documented dementia diagnosis compared with other payer types (66.2% for Medicare-Medicaid, 47.5% for Medicare fee-for-service, and 22.0% for Medicare Advantage) (Supplementary Table S2). Among beneficiaries diagnosed with dementia, 54.8% used home health care and 63.3% used hospice care. In comparison, among beneficiaries not diagnosed with dementia, 40.0% used home health care and 45.4% used hospice care (Table 2).

**Table 2.** Characteristics of 2019 Medicare Decedents by Dementia Diagnosis (Column %), *N* = 2,169,422

Variable	Overall (*n* = 2,169,422)	With dementia (*n* = 933,618)	No dementia (*n* = 1,235,804)
Hospice use, *n* (%)	1,151,966 (53.1)	591,272 (63.3)	560,694 (45.4)
Hospice days, median [IQR]	2 [0–19]	5 [0–40]	0 [0–10]
Age at death, mean (SD)	80.6 (10.7)	84.5 (9.4)	77.7 (10.7)
Age <68 at death	220,320 (10.2)	44,144 (4.7)	176,176 (14.3)
Female	1,123,377 (51.8)	550,717 (59.0)	572,660 (46.3)
Male	1,046,045 (48.2)	382,901 (41.0)	663,144 (53.66)
White, non-Hispanic	1,741,205 (80.3)	762,443 (81.7)	978,762 (79.2)
Black, non-Hispanic	218,740 (10.1)	92,425 (9.9)	126,315 (10.2)
Hispanic	148,630 (6.9)	53,879 (5.8)	94,751 (7.7)
Asian American/Pacific Islander	49,341 (2.3)	20,301 (2.2)	29,040 (2.4)
American Indian/Alaska Native	11,506 (0.5)	4570 (0.5)	6936 (0.6)
Medicare FFS only	973,802 (44.9)	462,167 (49.5)	511,635 (41.4)
Medicare FFS-Medicaid dual	380,375 (17.5)	251,697 (27.0)	128,678 (10.4)
Medicare advantage only	571,653 (26.4)	125,519 (13.4)	446,134 (36.1)
Medicare advantage dual	243,592 (11.2)	94,235 (10.1)	149,357 (12.1)
Neighborhood profile			
Urban, advantaged zip code	1,478,016 (68.1)	646,217 (69.2)	831,799 (67.3)
Urban, disadvantaged zip code	286,220 (13.2)	123,238 (13.2)	162,982 (13.2)
Rural, advantaged zip code	208,614 (9.6)	80,172 (8.6)	128,442 (10.4)
Rural, disadvantaged zip code	196,572 (9.1)	83,991 (9.0)	112,581 (9.1)
Count of CC, median [IQR]	6 [3–8]	8 [6–9]	5 [2–7]
Ischemic heart disease	1,309,399 (60.4)	676,223 (72.4)	633,176 (51.2)
Hypertension	1,747,319 (80.5)	859,143 (92.0)	888,176 (71.9)
Hyperlipidemia	1,568,459 (72.3)	788,334 (84.4)	780,125 (63.1)
Chronic kidney disease	1,170,501 (54.0)	616,681 (66.1)	553,820 (44.8)
Depression	983,424 (45.3)	590,212 (63.2)	393,212 (31.8)
Congestive heart failure	1,063,680 (49.0)	566,814 (60.7)	496,866 (40.2)
Diabetes	967,279 (44.6)	482,205 (51.6)	485,074 (39.3)
COPD	884,334 (40.8)	440,962 (47.2)	443,372 (35.9)
Stroke/TIA	564,405 (26.0)	359,671 (38.5)	204,734 (16.6)
Cancer	474,948 (21.9)	217,029 (23.3)	257,919 (20.9)
Acute myocardial infarction	244,620 (11.3)	127,840 (13.7)	116,780 (9.5)
End-stage renal disease	80,190 (3.7)	27,812 (3.0)	52,378 (4.2)
Hospitalizations, median [IQR]	2 [1–4]	3 [1–5]	2 [1–4]
SNF days, median [IQR]	0 [0–41]	24 [0–173]	0 [0–12]
≥100 SNF days	359,230 (16.6)	284,381 (30.5)	74,849 (6.1)
Home health care use, *n* (%)	1,005,681 (46.4)	511,691 (54.8)	493,990 (40.0)
None	1,163,741 (53.6)	421,927 (45.2)	741,814 (60.0)
Began in last year of life	380,905 (17.6)	154,057 (16.5)	226,848 (18.4)
Began before last year of life	624,776 (28.8)	357,634 (38.3)	267,142 (21.6)
Home health days, median [IQR]	0 [0–57]	16 [0–89]	0 [0–39]

*p* < 0.001 for all bivariate comparisons between groups with and without dementia.

COPD, chronic obstructive pulmonary disease; SNF, skilled nursing facility; TIA, transient ischemic attack.

### Adjusted regression analyses: home health care associated with a higher likelihood of hospice use

Home health care use in the last three years was associated with a higher likelihood of hospice use at the end of life (Table 3 and Supplementary Data Table S6). When individuals received home health care before the last year of life, they had 1.57 higher odds (95% confidence interval [CI] = 1.56–1.58) of hospice use than beneficiaries who had never received home health care. Analyses stratified by dementia diagnosis showed similar patterns with greater magnitude among beneficiaries without dementia. Medicare beneficiaries who received home health care only in the last year of life had 1.75 higher odds (95% CI = 1.74–1.77) of using hospice services compared with those who never received home health care, adjusting for sociodemographic, clinical, and regional variables. Sensitivity analyses, excluding beneficiaries who used home health after discharge from hospice (*n* = 671, 0.06% of our sample), and those who died in a nursing home (*n* = 121,669, 5.6% of sample) yielded comparable findings (Supplementary Tables S7a and S7b).

**Table 3.** Multivariable Logistic Regression of Hospice Use Overall and Stratified by Dementia Diagnosis

	All decedents		With dementia		Without dementia	
	*N* = 2,169,422		*n* = 933,618		*n* = 1,235,804	
	OR	95% CI	OR	95% CI	OR	95% CI
Home health use (Ref. = none)						
Started before last year of life	1.57	1.56–1.58	1.44	1.43–1.46	1.56	1.54–1.58
Started during last year of life	1.75	1.74–1.77	1.34	1.32–1.35	1.92	1.90–1.94
Race/ethnicity (Ref. = White)						
Black	0.67	0.67–0.68	0.70	0.69–0.71	0.66	0.65–0.67
Hispanic	0.81	0.80–0.82	0.83	0.81–0.85	0.82	0.80–0.83
AAPI	0.68	0.66–0.69	0.64	0.62–0.66	0.72	0.70–0.74
AIAN	0.74	0.71–0.77	0.70	0.66–0.74	0.76	0.72–0.81
Age at death (centered)	1.03	1.03–1.03	1.02	1.02–1.03	1.04	1.04–1.04
Age <68 at death	0.93	0.92–0.94	0.85	0.83–0.87	0.98	0.97–0.99
Female (Ref. = male)	1.20	1.19–1.21	1.17	1.15–1.18	1.20	1.19–1.21
Medicare FFS only	Ref.	Ref.	Ref.	Ref.	Ref.	Ref.
Medicare FFS-Medicaid dual	1.01	1.00–1.02	0.95	0.94–0.97	1.06	1.05–1.08
Medicare advantage only	1.33	1.32–1.34	1.31	1.29–1.33	1.39	1.38–1.41
Medicare advantage dual	1.21	1.20–1.22	1.00	0.98–1.02	1.31	1.29–1.33
Urban, advantaged zip code	Ref.	Ref.	Ref.	Ref.	Ref.	Ref.
Urban, disadvantaged zip	0.85	0.84–0.86	0.76	0.75–0.77	0.91	0.90–0.92
Rural, advantaged zip code	0.88	0.87–0.89	0.84	0.83–0.86	0.91	0.89–0.92
Rural, disadvantaged zip code	0.84	0.83–0.85	0.78	0.77–0.79	0.89	0.87–0.90
Chronic conditions						
Dementia	1.78	1.77–1.80	—	—	—	—
Ischemic heart disease	0.94	0.93–0.94	0.93	0.91–0.94	0.93	0.92–0.94
Hypertension	0.98	0.97–0.99	0.87	0.85–0.89	0.95	0.94–0.96
Hyperlipidemia	1.02	1.01–1.03	1.03	1.01–1.04	0.98	0.97–0.99
Chronic kidney disease	0.96	0.95–0.97	0.88	0.87–0.89	1.02	1.01–1.03
Depression	1.19	1.19–1.20	1.19	1.18–1.21	1.16	1.15–1.17
Congestive heart failure	0.88	0.88–0.90	0.84	0.83–0.85	0.93	0.92–0.94
Diabetes	0.89	0.88–0.89	0.92	0.91–0.93	0.87	0.87–0.88
COPD	0.92	0.92–0.93	0.86	0.85–0.87	0.99	0.98–0.99
Stroke/TIA	1.05	1.04–1.05	1.06	1.05–1.07	1.05	1.04–1.06
Cancer	1.40	1.37–1.43	1.12	1.09–1.16	1.63	1.59–1.68
AMI	0.81	0.80–0.82	0.85	0.84–0.86	0.78	0.77–0.79
End-stage renal disease	0.62	0.61–0.63	0.63	0.62–0.65	0.58	0.57–0.59
Health services used						
≥100 SNF days	0.90	0.89–0.91	0.87	0.86–0.88	0.96	0.94–0.97
Hospitalizations	1.01	1.01–1.01	0.98	0.98–0.98	1.04	1.04–1.04

Models were also adjusted for state and cancer subtype. The binary variable age at death <68 years was used to adjust for Medicare enrollment before age 65 associated with disability, based on the continuous enrollment inclusion criteria during the 2016–2019 look-back period.

AAPI, Asian American/Pacific Islander; AIAN, American Indian/Alaska Native; AMI, acute myocardial infarction; CI, confidence interval; COPD, chronic obstructive pulmonary disease; OR, odds ratio; SNF, skilled nursing facility; TIA, transient ischemic attack.

Supplementary Table S8a shows the adjusted odds ratios for hospice use from logistic regression with an interaction term between the timing of home health initiation and a dementia diagnosis. Supplementary Table S8b shows the predicted probabilities of hospice use generated from the logistic regression (Supplementary Table S8a). The association between the timing of home health initiation and hospice use was more pronounced among individuals without dementia. For instance, among those with dementia, individuals who began using home health in the last year had a 6% higher probability of hospice use compared with those who did not use home health. However, for those without dementia, individuals who began using home health in the last year had a 16% higher probability of hospice use compared with those who did not use home health. Persons who began using home health before the last year had a 13% higher probability of hospice use compared with those who did not use home health care.

The results of multinomial logistic regressions (Table 4) for hospice duration show the relative risk ratios (RRRs) for different duration categories after adjusting for beneficiaries' sociodemographic, geographic, and clinical factors. The outcome reference category was not using hospice care. The findings were stratified by dementia diagnosis. Among individuals with dementia, those who started using home health care before the last year versus those who did not use home health care had greater odds of using hospice for 1–2 days (RRR = 1.11, 95% CI = 1.09–1.14), 3–179 hospice days (RRR = 1.48, 95% CI = 1.50), and 180+ days (RRR = 1.68, 95% CI = 1.65–1.71). Those who started home health care in the last year of life versus those who did not use home health care had greater odds of using hospice for 1–2 days (RRR = 1.31, 95% CI = 1.28–1.35) and 3–179 days (RRR = 1.54, 95% CI = 1.52–1.56) but lower odds of using hospice for 180+ days (RRR = 0.32, 95% CI = 0.31–0.33). Among individuals without dementia, those who started using home health care before the last year versus those who did not use home health care had greater odds of using hospice for 1–2 days (RRR = 1.13, 95% CI = 1.11–1.16), 3–179 hospice days (RRR = 1.57, 95% CI = 1.55–1.58), and 180+ days (RRR = 2.81, 95% CI = 2.74–2.88). Those who started home health care in the last year of life versus those who did not use home health care had greater odds of using hospice for 1–2 days (RRR = 1.62, 95% CI = 1.59–1.65) and 3–179 days (RRR = 2.08, 95% CI = 2.05–2.10) but lower odds of using hospice for more than 180 days (RRR = 0.67, 95% CI = 0.64–0.70).

**Table 4.** Summary of Multinomial Logistic Regression of Receiving Hospice Care for Various Lengths of Time in Contrast to Not Receiving Hospice Care

Home health use (Ref. = none)	Model 2 RRR, 95% CI		
	1–2 Hospice days	3–179 Hospice days	180+ Hospice days
With dementia, *n* (%)	56,255 (6.0)	451,410 (48.4)	83,607 (9.0)
Started before last year	1.11 (1.09–1.14)	1.48 (1.47–1.50)	1.68 (1.65–1.71)
Started during last year of life	1.31 (1.28–1.35)	1.54 (1.52–1.56)	0.32 (0.31–0.33)
Without dementia, *n* (%)	74,310 (6.0)	446,230 (36.1)	40,154 (3.3)
Started before last year	1.13 (1.11–1.16)	1.57 (1.55–1.58)	2.81 (2.74–2.88)
Started during last year of life	1.62 (1.59–1.65)	2.08 (2.05–2.10)	0.67 (0.64–0.70)

Covariates included age, sex, race, type of health insurance, neighborhood profile, chronic conditions, hospitalizations, ≥100 days in skilled nursing facilities, and state. Comprehensive results from multinomial models with different cut points for length of hospice are given in Supplementary Tables S9 to S12.

All values where confidence intervals are displayed were clearly statistically significant.

RRR, relative risk ratio.

## Discussion

Among all Medicare decedents in 2019, nearly half used home health care in their last three years of life, with close to 30% starting before their final year. We found a strong association between home health care and hospice use at the end-of-life, and the effects were stronger in those without a diagnosis of dementia compared with those with dementia. This relationship may stem from the ability of home health care providers to identify terminal signs and support patients with chronic conditions, impacting decisions about end-of-life care.^29–31^ Financial incentives may also influence hospice referrals, given the higher per-diem rates of hospice care, especially from home health agencies owned by private equity firms and affiliated with hospice agencies. Although our findings indicate that earlier home health care can lead to longer hospice care, especially in dementia patients, the potential for financial motivations in end-of-life care transitions raise concerns.^31,32^

The observed association between the use of home health care and hospice services underscores the importance of continuity of care, suggesting that home health care can facilitate aging in place and reduce the need for burdensome care transitions.^20,33–35^ It also points to the need for further research on how home health and hospice agency ownership affects referral patterns and end-of-life care utilization.^36^

There are limitations, including not accounting for the severity of chronic conditions or the specifics of provider affiliations,^31,37^ and potential inaccuracies in dementia diagnoses in Medicare data.^38,39^ Nonetheless, this research fills a gap in understanding the timing of home health care initiation and its impact on hospice use.

The findings have clinical and policy implications, emphasizing home health care's role in enhancing hospice service use and the end-of-life care experience. They suggest the need for more resources in the home health sector and the development of staff competencies in end-of-life care. Moreover, quality measures could be expanded to include end-of-life care metrics. Future research should explore care transitions more deeply and assess the impact of organizational characteristics on hospice referrals.^32,40–42^ It should also investigate the role of home health in reducing racial and ethnic disparities in hospice care utilization.

This study contributes to literature by showing increased hospice use among home health recipients, consistent across demographics. With the trend toward aging in place, the significance of home health care in supporting end-of-life care is poised to grow, informing policies to bolster this sector.

## Ethical Compliance

The study was approved by Rutgers, The State University of New Jersey IRB.
