# Supplementary material for: Home Health Care and Hospice Use Among Medicare Beneficiaries With and Without a Diagnosis of Dementia
Source: J Palliat Med. 2024 Jun 22;27(6):776–83. doi: 10.1089/jpm.2023.0583 (PMC11310562; doi:10.1089/jpm.2023.0583)
Supplement: Supplementary Data [file jpm.2023.0583_suppl_data.pdf]

Supplemental Digital Content

Table S1. Characteristics of 2019 Medicare Decedents by Timing of Home Health Care Initiation During the Last Three Years of Life (row %), n=2,169,422

Table S2 Characteristics of 2019 Medicare Decedents by Dementia Diagnosis (row %), n=2,169,422

Table S3 Characteristics of 2019 Medicare Decedents with Dementia by Timing of Home Health Care Initiation During the Last Three Years of Life, *n* (column %), n=933,618

Table S4 Characteristics of 2019 Medicare Decedents without Dementia by Timing of Home Health Care Initiation During the Last Three Years of Life, *n* (column %) n=1,235,804

Table S5a. Sample Composition Based on the Home Health Care Use

Table S5b. Sample Composition Based on Alternate (Cumulative) Indicator of Home Health Care Use

Table S6. Logistic Regression Predicting Odds of Hospice Use from Alternate (Cumulative) Indicator of Home Health Use

Table S7a. Sensitivity Analysis 1. Results of logistic regression sensitivity analysis predicting the odds of hospice use, excluding individuals who used home health services after hospice (live discharge)

Table S7b. Sensitivity Analysis 2. Results of logistic regressions predicting the odds ratios of hospice use on a sample excluding individuals who died in a nursing home

Table S8a. Logistic Regression for Hospice use with Interaction (Home Health Use x Dementia)

Table S8b. Predicted Probability of Hospice use by Home Health Use and a Dementia Diagnosis

Table S9. Multinomial Logistic Regression of Hospice Use for Various Lengths of Time for Decedents with Dementia (1-2, 3-179, 180+ hospice days)

Table S10. Multinomial Logistic Regression of Hospice Use for Various Lengths of Time for Decedents with Dementia (1-7, 8-179, 180+ hospice days)

Table S11. Multinomial Logistic Regression of Hospice Use for Various Lengths of Time for Decedents without Dementia (1-2, 3-179, 180+ hospice days)

Table S12. Multinomial Logistic Regression of Hospice Use for Various Lengths of Time for Decedents without Dementia (1-7, 8-179, 180+ hospice days)

Text S1. Methodology of Calculating Home Health Care Days

Text S2. Methodology of Identifying Diagnosis of Alzheimer's Disease and Other Dementias

Table S1. Characteristics of 2019 Medicare Decedents by Timing of Home Health Care Initiation During the Last Three Years of Life (row %), n=2,169,422

Variable	None	Last Year	Prior to Last Year
Total sample, n (%)	1,163,741 (53.6)	380,905 (17.6)	624,776 (28.8)
Hospice use	539,036 (46.8)	231,564 (20.1)	381,366 (33.1)
Hospice days (median, IQR)	0 [0, 13]	3 [0, 18]	4 [0, 36]
Mean age at death (SD)	79.7 (11.0)	80.5 (10.0)	82.4 (10.4)
Age < 68 at death	134,580 (61.1)	33,617 (15.3)	52,123 (23.7)
Female	574,015 (51.1)	191,650 (17.1)	357,712 (31.8)
Male	589,726 (56.4)	189,255 (18.1)	267,064 (25.5)
White, non-Hispanic	923,954 (53.1)	310,973 (17.9)	506,278 (29.1)
Black, non-Hispanic	113,566 (51.9)	37,980 (17.4)	67,194 (30.7)
Hispanic	90,184 (60.7)	21,955 (14.8)	36,491 (24.6)
Asian American/Pacific Islander	28,948 (58.7)	8,321 (16.9)	12,072 (24.5)
American Indian/Alaska Native	7,089 (61.6)	1,676 (14.6)	2,741 (23.8)
Medicare and Medicaid			
Medicare Fee-for-Service only	492,453 (50.6)	191,565 (19.7)	289,794 (29.8)
Medicare FFS-Medicaid dual	215,547 (56.7)	45,781 (12.0)	119,047 (31.3)
Medicare Advantage only	316,613 (55.4)	110,226 (19.3)	144,814 (25.3)
Medicare Advantage dual	139,128 (57.1)	33,333 (13.7)	71,131 (29.2)
Urban, advantaged zip code	776,738 (52.6)	264,040 (17.9)	437,238 (29.6)
Urban, disadvantaged zip code	166,035 (58.0)	48,014 (16.8)	72,171 (25.2)
Rural, advantaged zip code	113,118 (54.2)	35,224 (16.9)	60,272 (28.9)
Rural, disadvantaged zip code	107,850 (54.9)	33,627 (17.1)	55,095 (28.0)
Chronic Conditions (CCs)			
Count of CCs (median, IQR)	5 [3,8]	6 [4,9]	7 [5,9]
ADRD/dementia	421,927 (45.2)	154,057 (16.5)	357,634 (38.3)
Ischemic Heart Disease	625,532 (47.8)	239,556 (18.3)	444,311 (33.9)
Hypertension	884,453 (50.6)	313,995 (18.0)	548,871 (31.4)
Hyperlipidemia	780,033 (49.7)	285,471 (18.2)	502,955 (32.1)
Chronic Kidney Disease	549,852 (47.0)	218,284 (18.7)	402,365 (34.4)
Depression	465,905 (47.4)	165,475 (16.8)	352,044 (35.8)
Congestive Heart Failure	481,654 (45.3)	196,384 (18.5)	385,642 (36.3)
Diabetes	460,591 (47.6)	174,372 (18.0)	332,316 (34.4)
COPD	408,958 (46.2)	162,230 (18.3)	313,146 (35.4)
Stroke/TIA	258,290 (45.8)	95,469 (16.9)	210,646 (37.3)
Cancer	230,270 (48.5)	98,407 (20.7)	146,275 (30.8)
Acute Myocardial Infarction	108,429 (44.3)	46,122 (18.9)	90,069 (36.8)
End-Stage Renal Disease	30,438 (38.0)	16,596 (20.7)	33,156 (41.4)
Hospitalizations (median, IQR)	1 [0, 3]	3 [2, 5]	4 [2, 7]
SNF days (median, IQR)	0 [0, 29]	0 [0, 27]	17 [0, 65]
≥ 100 SNF days	220,656 (61.4)	24,269 (6.8)	114,305 (31.8)

Note: Chi-squared tests for categorical variables and analyses of variance for continuous variables were all statistically significant with a *p*-value < 0.001. Health services utilization in the last three years was reported, except for hospice use within the last six months of life.

Table S2 Characteristics of 2019 Medicare Decedents Stratified by Dementia Diagnosis (row %)

Variable	Overall (n=2,169,422)	With Dementia (n=933,618)	No Dementia (n=1,235,804)
Hospice use (n, %)	1,151,966 (53.1)	591,272 (51.3)	560,694 (48.7)
Days in hospice (median, IQR)	2 [0, 19]	5 [0, 40]	0 [0, 10]
Mean Age at death (SD)	80.6 (10.7)	84.5 (9.4)	77.7 (10.7)
Age < 68 at death	220,320 (10.2)	44,144 (20.0)	176,176 (80.0)
Female	1,123,377 (51.8)	550,717 (49.0)	572,660 (51.0)
Male	1,046,045 (48.2)	382,901 (36.6)	663,144 (63.4)
White, non-Hispanic	1,741,205 (80.3)	762,443 (43.8)	978,762 (56.2)
Black, non-Hispanic	218,740 (10.1)	92,425 (42.3)	126,315 (57.8)
Hispanic	148,630 (6.9)	53,879 (36.3)	94,751 (63.8)
Asian American/Pacific Islander	49,341 (2.3)	20,301 (41.1)	29,040 (58.9)
American Indian/Alaska Native	11,506 (0.5)	4,570 (39.7)	6,936 (60.3)
Medicare Fee-for-Service only	973,802 (44.9)	462,167 (47.5)	511,635 (52.5)
Medicare FFS-Medicaid dual	380,375 (17.5)	251,697 (66.2)	128,678 (33.8)
Medicare Advantage only	571,653 (26.4)	125,519 (22.0)	446,134 (78.0)
Medicare Advantage dual	243,592 (11.2)	94,235 (38.7)	149,357 (61.3)
Urban, advantaged zip code	1,478,016 (68.1)	646,217 (43.7)	831,799 (56.3)
Urban, disadvantaged zip code	286,220 (13.2)	123,238 (43.1)	162,982 (56.9)
Rural, advantaged zip code	208,614 (9.6)	80,172 (38.4)	128,442 (61.6)
Rural, disadvantaged zip code	196,572 (9.1)	83,991 (42.7)	112,581 (57.3)
Count of CC (median, IQR)	6 [3, 8]	8 [6, 9]	5 [2, 7]
Ischemic Heart Disease	1,309,399 (60.4)	676,223 (51.6)	633,176 (48.4)
Hypertension	1,747,319 (80.5)	859,143 (49.2)	888,176 (50.8)
Hyperlipidemia	1,568,459 (72.3)	788,334 (50.3)	780,125 (49.7)
Chronic Kidney Disease	1,170,501 (54.0)	616,681 (52.7)	553,820 (47.3)
Depression	983,424 (45.3)	590,212 (60.0)	393,212 (40.0)
Congestive Heart Failure	1,063,680 (49.0)	566,814 (53.3)	496,866 (46.7)
Diabetes	967,279 (44.6)	482,205 (49.9)	485,074 (50.2)
COPD	884,334 (40.8)	440,962 (49.9)	443,372 (50.1)
Stroke/TIA	564,405 (26.0)	359,671 (63.7)	204,734 (36.3)
Cancer	474,948 (21.9)	217,029 (45.7)	257,919 (54.3)
Acute Myocardial Infarction	244,620 (11.3)	127,840 (52.3)	116,780 (47.7)
End-Stage Renal Disease	80,190 (3.7)	27,812 (34.7)	52,378 (65.3)
Health Services Use			
Hospitalizations (median, IQR)	2 [1, 4]	3 [1, 5]	2 [1, 4]
SNF days (median, IQR)	0 [0, 41]	24 [0, 173]	0 [0, 12]
≥ 100 SNF days	359,230 (16.6)	284,381 (79.2)	74,849 (20.8)
Home Health Care Use (n, %)	1,005,681 (46.4)	511,691 (50.9)	493,990 (49.1)
None	1,163,741 (53.6)	421,927 (36.3)	741,814 (63.7)
Began in Last Year of Life	380,905 (17.6)	154,057 (40.4)	226,848 (59.6)
Began Prior to Last Year of Life	624,776 (28.8)	357,634 (57.2)	267,142 (42.8)
Home Health Days (median, IQR)	0 [0, 57]	16 [0, 89]	0 [0, 39]

Note: *p*-value < 0.001 for all bivariate comparisons between groups with and without a diagnosis of dementia.

Table S3 Characteristics of 2019 Medicare Decedents with Dementia by Timing of Home Health Care Initiation During the Last Three Years of Life (column %), n=933,618

	None (n= 421,927)	Last Year (n= 154,057)	Prior to Last Year (n= 357,634)
Hospice use, n (%)	250,218 (59.3)	101,722 (66.0)	239,332 (66.9)
Hospice days (median, IQR)	4 [0, 37]	5 [0, 25]	7 [0,54]
Mean age at death (SD)	84.5 (9.7)	83.9 (9.0)	84.8 (9.1)
Age < 68 at death	22,034 (5.2)	6,627 (4.3)	15,483 (4.3)
Female	253,686 (60.1)	82,551 (53.6)	214,480 (60.0)
Male	168,241 (39.9)	71,506 (46.4)	143,154 (40.0)
White, non-Hispanic	343,521 (81.4)	127,029 (82.5)	291,893 (81.6)
Black, non-Hispanic	40,924 (9.7)	14,666 (9.5)	36,835 (10.3)
Hispanic	25,210 (6.0)	8,298 (5.4)	20,371 (5.7)
Asian American/Pacific Islander	9,768 (2.3)	3,408 (2.2)	7,125 (2.0)
American Indian/Alaska Native	2,504 (0.6)	656 (0.4)	1,410 (0.4)
Medicare Fee-for-Service only	177,797 (42.1)	94,279 (61.2)	190,091 (53.2)
Medicare FFS-Medicaid dual	144,912 (34.4)	23,668 (15.4)	83,117 (23.2)
Medicare Advantage only	44,723 (10.6)	26,684 (17.3)	54,112 (15.1)
Medicare Advantage dual	54,495 (12.9)	9,426 (6.1)	30,314 (8.5)
Urban, advantaged zip code	281,179 (66.6)	109,376 (71.0)	255,662 (71.5)
Urban, disadvantaged zip code	64,005 (15.2)	18,818 (12.2)	40,415 (11.3)
Rural, advantaged zip code	36,512 (8.7)	12,596 (8.2)	31,064 (8.7)
Rural, disadvantaged zip code	40,231 (9.5)	13,267 (8.6)	30,493 (8.5)
Chronic Conditions (CC)			
Count of CCs (median, IQR)	7 [5,9]	8 [6,9]	8 [6,10]
Ischemic Heart Disease	289,387 (68.6)	110,802 (71.9)	276,034 (77.2)
Hypertension	385,828 (91.4)	139,809 (90.8)	333,506 (93.3)
Hyperlipidemia	347,301 (82.3)	129,960 (84.4)	311,073 (87.0)
Chronic Kidney Disease	261,321 (61.9)	102,413 (66.5)	252,947 (70.7)
Depression	266,171 (63.1)	86,386 (56.1)	237,655 (66.5)
Congestive Heart Failure	236,559 (56.1)	91,527 (59.4)	238,728 (66.8)
Diabetes	205,746 (48.8)	76,781 (49.9)	199,678 (55.8)
COPD	182,158 (43.2)	70,434 (45.7)	188,370 (52.7)
Stroke/TIA	153,711 (36.4)	54,770 (35.6)	151,190 (42.3)
Cancer	90,489 (21.5)	39,533 (25.7)	87,007 (24.3)
Acute Myocardial Infarction	49,176 (11.7)	22,097 (14.3)	56,567 (15.8)
End-Stage Renal Disease	8,393 (2.0)	5,237 (3.4)	14,182 (4.0)
Health Services Use			
Hospitalizations (median, IQR)	2 [0,4]	3 [2,5]	4 [2,7]
SNF days (median, IQR)	37 [0, 712]	11 [0, 42]	26 [0, 95]
≥ 100 SNF days	182,694 (43.3)	15,777 (10.2)	85,910 (24.0)

Note: Chi-squared tests for categorical variables and analyses of variance for continuous variables were all statistically significant with a p-value < 0.001. Health services utilization in the last three years was reported, except for hospice use within the last six months of life.

Table S4 Characteristics of 2019 Medicare Decedents without Dementia by Timing of Home Health Care Initiation During the Last Three Years of Life (column %), n=1,235,804

	None (n=741,814)	Last Year (n=226,848)	Prior to Last Year (n=267,142)
Hospice use, n (%)	288,818 (38.9)	129,842 (57.2)	142,034 (53.2)
Hospice days (median, IQR)	0 [0, 7]	2 [0, 14]	2[0,19]
Mean age at death (SD)	77.0 (10.7)	78.3 (10.1)	79.1 (11.0)
Age < 68 at death	112,546 (15.2)	26,990 (11.9)	36,640 (13.7)
Female	320,329 (43.2)	109,099 (48.1)	143,232 (53.6)
Male	421,485 (56.8)	117,749 (51.9)	123,910 (46.4)
White, non-Hispanic	580,443 (78.3)	183,944 (81.1)	214,385 (80.3)
Black, non-Hispanic	72,642 (9.8)	23,314 (10.3)	30,359 (11.4)
Hispanic	64,974 (8.8)	13,657 (6.0)	16,120 (6.0)
Asian American/Pacific Islander	19,180 (2.6)	4,913 (2.2)	4,947 (1.9)
American Indian/Alaska Native	4,585 (0.6)	1,020 (0.5)	1,331 (0.5)
Medicare Fee-for-Service only	314,656 (42.4)	97,289 (42.9)	99,693 (37.3)
Medicare FFS-Medicaid dual	70,635 (9.5)	22,113 (9.8)	35,930 (13.5)
Medicare Advantage only	271,890 (36.7)	83,542 (36.8)	90,702 (34.0)
Medicare Advantage dual	84,633 (11.4)	23,907 (10.5)	40,817 (15.3)
Urban, advantaged zip code	495,559 (66.8)	154,664 (68.2)	181,576 (68.0)
Urban, disadvantaged zip code	102,030 (13.8)	29,196 (12.9)	31,756 (11.9)
Rural, advantaged zip code	76,606 (10.3)	22,628 (10.0)	29,208 (10.9)
Rural, disadvantaged zip code	67,619 (9.1)	20,360 (9.0)	24,602 (9.2)
Chronic Conditions (median, IQR)	4 [1,6]	5 [3, 7]	6 [3, 8]
Ischemic Heart Disease	336,145 (45.3)	128,754 (56.8)	168,277 (63.0)
Hypertension	498,625 (67.2)	174,186 (76.8)	215,365 (80.6)
Hyperlipidemia	432,732 (58.3)	155,511 (68.6)	191,882 (71.8)
Chronic Kidney Disease	288,541 (38.9)	115,871 (51.1)	149,418 (55.9)
Depression	199,734 (26.9)	79,089 (34.9)	114,389 (42.8)
Congestive Heart Failure	245,095 (33.0)	104,857 (46.2)	146,914 (55.0)
Diabetes	254,845 (34.4)	97,591 (43.0)	132,638 (49.7)
COPD	226,800 (30.6)	91,796 (40.5)	124,776 (46.7)
Stroke/TIA	104,579 (14.1)	40,699 (17.9)	59,456 (22.3)
Cancer	139,781 (18.8)	58,870 (26.0)	59,268 (22.2)
Acute Myocardial Infarction	59,253 (8.0)	24,025 (10.6)	33,502 (12.5)
End-Stage Renal Disease	22,045 (3.0)	11,359 (5.0)	18,974 (7.1)
Health Services Use			
Hospitalizations (median, IQR)	1 [0,3]	3 [2,5]	4 [2,7]
SNF days (median, IQR)	0 [0, 0]	0 [0, 20]	7 [0, 38]
≥ 100 SNF days	37,962 (5.1)	8,492 (3.7)	28,395 (10.6)

Note: Chi-squared tests for categorical variables and analyses of variance for continuous variables were all statistically significant with a p-value < 0.001. Health services utilization in the last three years was reported, except for hospice use within the last six months of life.

Table S5a. Sample Composition by Home Health Care Use During Last Three Years of Life

	Third-to-Last Year of Life	Second-to-Last Year of Life	Last Year of Life	N
No Home Health Care Use				1,163,741 (53.6%)
Started in Last Year of Life				380,905 (17.6%)
Started Prior to Last Year				213,087 (9.8%)
				130,919 (6.0%)
				130,145 (6.0%)
				60,294 (2.8%)
				55,568 (2.6%)
				34,763 (1.6%)

Table S5b. Sample Composition for Alternative (Cumulative) Indicator of Home Health Care Use

Group	Y3	Y2	Y1	
1: No HH				1,163,741 (53.6%)
2: Y1 only				380,905 (17.6%)
3: Y2 and Y1				213,087 (9.8%)
4: Y3, Y2, & Y1				130,145 (6.0%)
5: Other (Y3 & Y1, Y2 or Y3 only)				281,544 (13.0%)

Table S6. Logistic Regression Predicting Odds of Hospice Use from Alternate (Cumulative) Indicator of Home Health Use

Outcome: hospice use in the last 6 months of life (0/1) – compare with main findings in Table 3

New HH variable	With Dementia		Without Dementia	
	OR	95% CI	OR	95% CI
Group 1 (None)	Ref.		Ref.	
Group 2 (Y1)	1.34	1.32-1.35	1.92	1.90-1.94
Group 3 (Y1+Y2)	1.44	1.42-1.47	1.69	1.66-1.71
Group 4 (Y1+Y2+Y3)	1.42	1.40-1.45	1.51	1.48-1.54
Group 5 (The rest)	1.45	1.43-1.47	1.50	1.48-1.54

Sensitivity Analyses

Table S7a. Sensitivity Analysis 1. Results of logistic regression sensitivity analysis predicting the odds of hospice use, excluding individuals who used home health services after hospice (live discharge)

Individuals who used home health care after hospice (live discharge): 671
Individuals with dementia who used home health after hospice: 381
Individuals without dementia who used home health after hospice: 290

Original Model	All Decedents		With Dementia		Without Dementia	
	n= 2,169,422		n=933,618		n=1,235,804	
	OR	95% CI	OR	95% CI	OR	95% CI
Home Health Use (Ref = none)						
Started prior to last year	1.57	1.56-1.58	1.44	1.43-1.46	1.56	1.54-1.58
Started during last year of life	1.75	1.74-1.77	1.34	1.32-1.35	1.92	1.90-1.94
Sensitivity Analyses	Decedents excluding those who used home health after hospice		Decedents with dementia excluding those who used home health after hospice		Decedents without dementia excluding those who used home health after hospice	
	N=2,168,751		N=933,237		N=1,235,514	
Home Health Use (Ref = none)						
Started prior to last year	1.57	1.56-1.58	1.44	1.43-1.46	1.56	1.54-1.57
Started during last year of life	1.75	1.74-1.76	1.33	1.32-1.35	1.92	1.90-1.94

Table S7b. Sensitivity Analysis 2. Results of logistic regressions predicting the odds ratios of hospice use on a sample excluding individuals who died in a nursing home

Individuals who died in a nursing home: 121,669
Individuals with dementia who died in a nursing home: 83,354
Individuals without dementia who died in a nursing home: 38,315

Original Model	All Decedents		With Dementia		Without Dementia	
	n= 2,169,422		n=933,618		n=1,235,804	
	OR	95% CI	OR	95% CI	OR	95% CI
Home Health Use (Ref = none)						
Started before last year	1.57	1.56-1.58	1.44	1.43-1.46	1.56	1.54-1.58
Started in last year of life	1.75	1.74-1.77	1.34	1.32-1.35	1.92	1.90-1.94
Sensitivity Analyses	Decedents excluding those who died in nursing home		Decedents with dementia excluding those who died in nursing home		Decedents without dementia excluding those who died in nursing home	
	N=2,047,753		N=850,264		N=1,197,489	
Home Health Use (Ref = none)						
Started before last year	1.57	1.55-1.58	1.41	1.40-1.43	1.58	1.56-1.59
Started in last year of life	1.77	1.76-1.79	1.30	1.28-1.32	1.95	1.93-1.97

Table S8a. Logistic Regression for Hospice Use with Interaction (Home Health Use x Dementia)

	All Decedents (n=2,169,422)	
	Adjusted Odds Ratio	95% CI
Home Health Use (Ref = none)		
Started prior to last year	1.78	1.76-1.80
Started in last year of life	2.08	2.06-2.10
Dementia Diagnosis	2.14	2.12-2.16
Home Heath ## Dementia		
Started prior to the last year # Dementia	0.75	0.74-0.76
Started in last year of life # Dementia	0.62	0.61-0.63

Table S8b. Predicted Probability of Hospice Use by Home Health Use and a Dementia Diagnosis

	Predicted probability of hospice use	95% CI
No HH	0.48	0.48-0.48
Started HH prior to Last Year	0.58	0.58-0.59
Started HH in Last Year	0.60	0.60-0.60
Without Dementia	0.48	0.47-0.48
With Dementia	0.61	0.61-0.61
No HH # Dementia	0.58	0.58-0.58
HH Started prior to last year # Dementia	0.64	0.64-0.65
HH Started in last year # Dementia	0.64	0.64-0.64
No HH # Without Dementia	0.41	0.41-0.41
HH Started prior to last year # Without Dementia	0.54	0.54-0.54
HH Started in last year # Without Dementia	0.57	0.57-0.58

Table S9. Multinomial Logistic Regression of Hospice Use for Various Lengths of Time for Decedents with Dementia (1-2, 3-179, 180+ hospice days)

	1-2 Hospice Days Vs. No Hospice		3-179 Hospice Days vs. No Hospice		180+ Hospice Days vs. No Hospice	
	RRR	95% CI	RRR	95% CI	RRR	95% CI
Home Health Use (Ref= none)						
Started prior to last year	1.11	1.09-1.14	1.48	1.47-1.50	1.68	1.65-1.71
Started in last year of life	1.31	1.28-1.35	1.54	1.52-1.56	0.32	0.31-0.33
Race/Ethnicity (Ref = white)						
Black	0.58	0.56-0.60	0.73	0.72-0.74	0.64	0.62-0.66
Hispanic	0.81	0.77-0.84	0.85	0.83-0.87	0.76	0.73-0.79
AAPI	0.71	0.66-0.77	0.66	0.63-0.68	0.53	0.50-0.57
AIAN	0.70	0.61-0.80	0.72	0.67-0.76	0.60	0.53-0.69
Age at death (centered)	1.02	1.01-1.02	1.02	1.02-1.02	1.03	1.03-1.04
Age < 68 at death	0.85	0.81-0.90	0.81	0.79- 0.84	0.96	0.91-1.02
Female (Ref = male)	0.98	0.96-1.00	1.16	1.14-1.17	1.40	1.37-1.42
Medicare Fee-for-Service only	Ref.	Ref.	Ref.	Ref.	Ref.	Ref.
Medicare FFS-Medicaid dual	0.89	0.86-0.91	0.93	0.92-0.94	1.22	1.19-1.24
Medicare Advantage only	1.09	1.06-1.13	1.32	1.30-1.34	1.42	1.38-1.46
Medicare Advantage dual	0.83	0.80-0.87	1.00	0.98-1.01	1.19	1.15-1.22
Urban, advantaged zip code	Ref.	Ref.	Ref.	Ref.	Ref.	Ref.
Urban, disadvantaged zip code	0.79	0.77-0.81	0.77	0.76-0.78	0.69	0.67-0.71
Rural, advantaged zip code	0.87	0.85-0.90	0.85	0.84-0.87	0.77	0.75-0.80
Rural, disadvantaged zip code	0.81	0.78-0.84	0.79	0.78-0.81	0.71	0.69-0.74
Chronic Conditions						
Ischemic Heart Disease	0.97	0.95-0.99	0.93	0.91-0.94	0.91	0.90-0.93
Hypertension	0.89	0.85-0.93	0.86	0.84-0.88	0.93	0.90-0.96
Hyperlipidemia	1.00	0.97-1.03	1.05	1.03-1.06	0.95	0.92-0.97
Chronic Kidney Disease	1.07	1.05-1.10	0.90	0.89-0.91	0.76	0.74-0.77
Depression	0.97	0.95-0.99	1.17	1.16-1.19	1.54	1.51-1.56
Congestive Heart Failure	1.02	0.99-1.04	0.82	0.81-0.83	0.87	0.85-0.89
Diabetes	0.94	0.93-0.96	0.92	0.91-0.93	0.91	0.89-0.92
COPD	0.96	0.94-0.99	0.84	0.84-0.85	0.92	0.90-0.93
Stroke/TIA	1.06	1.04-1.08	1.05	1.04-1.06	1.12	1.10-1.14
Cancer	1.10	1.03-1.17	1.14	1.10-1.18	1.04	0.97-1.10
AMI	0.90	0.87-0.92	0.85	0.84-0.86	0.79	0.77-0.82
End-Stage Renal Disease	0.84	0.80-0.88	0.62	0.61-0.64	0.12	0.10-0.14
Health Services Used						
≥ 100 SNF days	1.01	0.98-1.03	0.90	0.89- 0.92	0.56	0.55-0.58
Hospitalizations	1.03	1.02-1.03	0.99	0.99-0.99	0.80	0.80-0.80

Note: RRR= relative risk ratio. 95% CI=95% Confidence Interval. Models were adjusted for state and cancer subtype.

Table S10. Multinomial Logistic Regression of Hospice Use for Various Lengths of Time for Decedents with Dementia (1-7, 8-179, 180+ hospice days)

	1-7 Hospice Days vs. No Hospice		8-179 Hospice Days vs. No Hospice		180+ Hospice Days vs. No Hospice	
	RRR	95% CI	RRR	95% CI	RRR	95% CI
Home Health Use (Ref= none)						
Started prior to last year	1.19	1.17-1.20	1.598	1.58-1.62	1.69	1.66-1.72
Started in last year of life	1.40	1.37-1.42	1.586	1.56-1.61	0.32	0.31-0.33
Race/Ethnicity (Ref = white)						
Black	0.61	0.60-0.63	0.77	0.76-0.79	0.64	0.62-0.66
Hispanic	0.79	0.77-0.81	0.85	0.83-0.87	0.76	0.73-0.79
AAPI	0.719	0.69-0.75	0.63	0.61-0.65	0.53	0.50-0.57
AIAN	0.70	0.64-0.76	0.72	0.67- 0.78	0.60	0.53-0.69
Age at death (centered)	1.02	1.01-1.02	1.02	1.02-1.02	1.03	1.03-1.04
Age < 68 at death	0.83	0.80-0.86	0.81	0.79-0.84	0.96	0.91-1.02
Female (Ref = male)	1.03	1.02-1.05	1.20	1.18-1.21	1.40	1.37-1.42
Medicare Fee-for-Service only	Ref.	Ref.	Ref.	Ref.	Ref.	Ref.
Medicare FFS-Medicaid dual	0.89	0.87-0.90	0.95	0.94-0.96	1.22	1.19-1.25
Medicare Advantage only	1.17	1.14-1.19	1.36	1.34-1.39	1.42	1.39-1.46
Medicare Advantage dual	0.89	0.87-0.91	1.03	1.01-1.05	1.19	1.16-1.23
Urban, advantaged zip code	Ref.	Ref.	Ref.	Ref.	Ref.	Ref.
Urban, disadvantaged zip code	0.79	0.77-0.80	0.76	0.75-0.78	0.69	0.67-0.71
Rural, advantaged zip code	0.87	0.85-0.89	0.85	0.83-0.86	0.77	0.75-0.80
Rural, disadvantaged zip code	0.81	0.79-0.83	0.79	0.77-0.80	0.71	0.69-0.74
Chronic Conditions						
Ischemic Heart Disease	0.96	0.94-0.97	0.92	0.91-0.93	0.91	0.90-0.93
Hypertension	0.85	0.83-0.87	0.86	0.85 0.88	0.93	0.90-0.96
Hyperlipidemia	1.02	1.00-1.04	1.05	1.04-1.07	0.95	0.93-0.97
Chronic Kidney Disease	1.02	1.00-1.03	0.87	0.85-0.88	0.76	0.74-0.77
Depression	1.02	1.01-1.04	1.23	1.21-1.24	1.54	1.51-1.57
Congestive Heart Failure	0.91	0.90-0.93	0.80	0.79-0.81	0.87	0.85-0.89
Diabetes	0.93	0.91-0.94	0.92	0.91-0.93	0.91	0.89-0.92
COPD	0.89	0.88-0.90	0.84	0.83-0.85	0.92	0.90-0.93
Stroke/TIA	1.08	1.08-1.10	1.03	1.02-1.04	1.12	1.10-1.14
Cancer	1.12	1.08-1.17	1.14	1.10-1.18	1.04	0.97-1.10
AMI	0.88	0.87-0.90	0.84	0.82-0.85	0.79	0.77-0.82
End-Stage Renal Disease	0.97	0.94-0.99	0.44	0.43-0.46	0.12	0.10-0.14
Health Services Used						
≥ 100 SNF days	0.95	0.94-0.97	0.90	0.89- 0.91	0.56	0.55-0.58
Hospitalizations	1.02	1.02-1.02	0.98	0.98-0.98	0.80	0.80-0.80

Table S11. Multinomial Logistic Regression of Hospice Use for Various Lengths of Time for Decedents without Dementia (1-2, 3-179, 180+ hospice days)

	1-2 Hospice Days Vs. No Hospice		3-179 Hospice Days vs. No Hospice		180+ Hospice Days vs. No Hospice	
	RRR	95% CI	RRR	95% CI	RRR	95% CI
Home Health Use (Ref = none)						
Started prior to last year	1.13	1.11-1.16	1.57	1.55-1.58	2.81	2.74-2.88
Started in last year of life	1.62	1.59-1.65	2.08	2.05-2.10	0.67	0.64-0.70
Race/Ethnicity (Ref = white)						
Black	0.57	0.56- 0.59	0.68	0.67-0.69	0.56	0.54-0.59
Hispanic	0.79	0.76-0.82	0.82	0.81-0.84	0.79	0.75-0.83
AAPI	0.75	0.71-0.80	0.73	0.71-0.75	0.60	0.55-0.65
AIAN	0.78	0.70- 0.88	0.77	0.73-0.82	0.67	0.57-0.79
Age at death (centered)	1.03	1.03-1.03	1.04	1.04-1.04	1.07	1.07-1.07
Age < 68 at death	0.88	0.85-0.91	0.96	0.94-0.97	1.55	1.47-1.62
Female (Ref = Male)	1.11	1.09-1.13	1.20	1.19-1.21	1.35	1.32-1.39
Medicare Fee-for-Service only	Ref.	Ref.	Ref.	Ref.	Ref.	Ref.
Medicare FFS-Medicaid dual	0.94	0.92-0.97	1.04	1.03-1.06	1.63	1.57-1.70
Medicare Advantage only	1.47	1.44-1.50	1.38	1.37-1.40	1.48	1.44-1.53
Medicare Advantage dual	1.26	1.22-1.29	1.30	1.28-1.32	1.66	1.59-1.72
Urban, advantaged zip code	Ref.	Ref.	Ref.	Ref.	Ref.	Ref.
Urban, disadvantaged zip code	0.87	0.85-0.89	0.92	0.91-0.93	0.87	0.84-0.90
Rural, advantaged zip code	0.92	0.89-0.94	0.91	0.89-0.92	0.89	0.86-0.93
Rural, disadvantaged zip code	0.85	0.83-0.88	0.90	0.88-0.91	0.84	0.81-0.88
Chronic Conditions						
Ischemic Heart Disease	0.96	0.94-0.98	0.92	0.91-0.93	0.99	0.96-1.02
Hypertension	0.97	0.94-0.99	0.95	0.93-0.96	0.94	0.91-0.97
Hyperlipidemia	0.96	0.94-0.98	1.00	0.99-1.01	0.81	0.78-0.83
Chronic Kidney Disease	1.21	1.18-1.23	1.01	1.00-1.02	0.86	0.83-0.88
Depression	0.96	0.94-0.98	1.17	1.16-1.18	1.51	1.47-1.55
Congestive Heart Failure	1.14	1.12-1.16	0.88	0.87-0.89	1.15	1.12-1.18
Diabetes	0.88	0.86-0.89	0.87	0.86-0.88	0.86	0.84-0 .89
COPD	1.03	1.01-1.05	0.95	0.94-0.96	1.46	1.43- 1.50
Stroke/TIA	1.17	1.15-1.19	1.03	1.02-1.04	1.05	1.02-1.08
Cancer	1.39	1.32-1.46	1.69	1.64-1.74	1.52	1.41-1.63
AMI	0.80	0.78-0.82	0.78	0.77-0.79	0.82	0.79-0.85
End-Stage Renal Disease	0.79	0.76-0.82	0.55	0.54-0.57	0.14	0.12-0.17
Health Services Used						
≥ 100 SNF days	0.98	0.95-1.01	0.97	0.96-0.99	0.74	0.71-0.78
Hospitalizations	1.08	1.08-1.09	1.05	1.05-1.05	0.83	0.82-0.83

Note: RRR= relative risk ratio. 95% CI=95% Confidence Interval. Models were adjusted for state and cancer subtype.

Table S12. Multinomial Logistic Regression of Hospice Use for Various Lengths of Time for Decedents without Dementia (1-7, 8-179, 180+ hospice days)

	1-7 Hospice Days Vs. No Hospice		8-179 Hospice Days vs. No Hospice		180+ Hospice Days vs. No Hospice	
	RRR	95% CI	RRR	95% CI	RRR	95% CI
Home Health Use (Ref = none)						
Started prior to last year	1.225	1.21-1.24	1.72	1.70-1.74	2.82	2.75-2.89
Started in last year of life	1.828	1.80-1.85	2.14	2.11-2.16	0.67	0.64-0.70
Race/Ethnicity (Ref = white)						
Black	0.60	0.59-0.61	0.71	0.70-0.72	0.56	0.54-0.59
Hispanic	0.78	0.76-0.80	0.85	0.83-0.86	0.79	0.75-0.83
AAPI	0.71	0.68-0.74	0.74	0.72-0.77	0.60	0.55-0.65
AIAN	0.77	0.72- 0.83	0.77	0.72-0.82	0.67	0.57-0.79
Age at death (centered)	1.03	1.03-1.03	1.04	1.04-1.04	1.07	1.07-1.07
Age < 68 at death	0.89	0.87-0.91	0.99	0.97-1.00	1.55	1.47-1.62
Female (Ref = Male)	1.13	1.11-1.14	1.23	1.21-1.24	1.36	1.33-1.39
Medicare Fee-for-Service only	Ref.	Ref.	Ref.	Ref.	Ref.	Ref.
Medicare FFS-Medicaid dual	0.94	0.92-0.96	1.09	1.07-1.11	1.64	1.57-1.70
Medicare Advantage only	1.44	1.42-1.46	1.36	1.35-1.38	1.48	1.44-1.53
Medicare Advantage dual	1.26	1.23-1.28	1.32	1.30-1.34	1.66	1.60-1.72
Urban, advantaged zip code	Ref.	Ref.	Ref.	Ref.	Ref.	Ref.
Urban, disadvantaged zip code	0.90	0.88-0.91	0.92	0.91-0.94	0.87	0.84-0.90
Rural, advantaged zip code	0.92	0.90-0.93	0.90	0.89-0.92	0.89	0.86-0.93
Rural, disadvantaged zip code	0.87	0.85-0.89	0.90	0.89-0.92	0.84	0.81-0.88
Chronic Conditions						
Ischemic Heart Disease	0.94	0.93- 0.95	0.92	0.91-0.93	0.99	0.96-1.02
Hypertension	0.96	0.94- 0.98	0.94	0.93-0.95	0.94	0.91-0.97
Hyperlipidemia	0.99	0.97-1.00	1.00	0.99-1.01	0.81	0.78-0.83
Chronic Kidney Disease	1.16	1.14-1.17	0.96	0.95-0.97	0.86	0.83-0.88
Depression	1.03	1.02-1.05	1.22	1.21-1.23	1.51	1.48-1.55
Congestive Heart Failure	0.99	0.98-1.01	0.86	0.85-0.87	1.15	1.12-1.18
Diabetes	0.88	0.87-0.89	0.87	0.86-0.88	0.86	0.84-0.89
COPD	0.96	0.95-0.97	0.96	0.95-0.97	1.46	1.43- 1.50
Stroke/TIA	1.15	1.14-1.17	0.98	0.97-0.99	1.05	1.02-1.08
Cancer	1.435	1.38-1.49	1.80	1.75-1.86	1.52	1.41- 1.63
AMI	0.78	0.77-0.80	0.78	0.77-0.80	0.82	0.79- 0.85
End-Stage Renal Disease	0.83	0.81-0.86	0.40	0.38-0.41	0.14	0.12-0.16
Health Services Used						
≥ 100 SNF days	0.99	0.97-1.01	0.97	0.94-0.98	0.74	0.70-0.78
Hospitalizations	1.08	1.08-1.08	1.03	1.03-1.03	0.83	0.82-0.83

Text S1. Methodology of Calculating Home Health Care Days

The main independent variable was a three-category indicator of the timing of home health use: never used home health care in the last three years of life, began using home health care in the last year, and used prior to the last year during the last three years. To calculate home health days, we retained assessment dates and assessment reasons from the OASIS file for our sample during the study period of 2016 to 2019. After sorting the assessment dates in reverse chronological order and creating a lag variable that preserved the date for the next assessment date alongside the prior assessment date, we calculated the number of home health days by subtracting the next assessment dates from the prior assessment dates. Additional rules were employed to address specific cases identified in the data. For assessments with reasons indicating the start of care or resumption of care, if the calculated days exceeded 60 days, we assigned a value of 60 days. In the case of reassessments for follow-up, if the calculated days exceeded 65 days, we capped it at a value of 65 days. We applied this rule because it would be unreasonable to assume that patients received home health care for that long period of time, and the unexpectedly long calculated home health days may be attributed to a gap in the continuity of home health care between those assessments.

Text S2. Methodology of Identifying Diagnosis of Alzheimer's Disease and Other Dementias

Alzheimer's disease and related dementia (ADRD) were identified using the International Classification of Diseases, Tenth Revision (ICD-10) and Ninth Revision (ICD-9) diagnosis codes (331.0, 331.11, 331.19, 331.7, 331.2, 290.0, 290.11, 290.13, 290.20, 290.21, 290.3, 290.40, 290.41, 290.42, 290.43, 294.0, 294.10, 290.11, 294.20, 294.21, 294.8, 797, G30.0, G30.1, G30.8, G30.9, F01.50, F01.51, F02.80, F02.81, F03.90, F03.91, F04, G13.8, F05, F06.1, F06.8, G30.0, G30.1, G30.8, G30.9, G31.1, G31.2, G31.01, G31.09, G94, R41.81, and R54). We augmented the Master Beneficiary Summary File Chronic Conditions segment by additionally leveraging available diagnostic information from MedPAR, OASIS, and MDS to identify if patients were diagnosed with dementia.
