# Supplementary material for: Home Health Care and Hospice Use Among Medicare Beneficiaries With and Without a Diagnosis of Dementia
Source: J Palliat Med. 2024 Jun 22;27(6):776–83. doi: 10.1089/jpm.2023.0583 (PMC11310562; doi:10.1089/jpm.2023.0583)
Supplement: Supplementary Table S1 [file jpm.2023.0583_suppl_tables1.pdf]

Table S1. Characteristics of 2019 Medicare Decedents by Timing of Home Health Care Initiation During the Last Three Years of Life (row %), n=2,169,422

Variable	None	Last Year	Prior to Last Year
Total sample, n (%)	1,163,741 (53.6)	380,905 (17.6)	624,776 (28.8)
Hospice use	539,036 (46.8)	231,564 (20.1)	381,366 (33.1)
Hospice days (median, IQR)	0 [0, 13]	3 [0, 18]	4 [0, 36]
Mean age at death (SD)	79.7 (11.0)	80.5 (10.0)	82.4 (10.4)
Age < 68 at death	134,580 (61.1)	33,617 (15.3)	52,123 (23.7)
Female	574,015 (51.1)	191,650 (17.1)	357,712 (31.8)
Male	589,726 (56.4)	189,255 (18.1)	267,064 (25.5)
White, non-Hispanic	923,954 (53.1)	310,973 (17.9)	506,278 (29.1)
Black, non-Hispanic	113,566 (51.9)	37,980 (17.4)	67,194 (30.7)
Hispanic	90,184 (60.7)	21,955 (14.8)	36,491 (24.6)
Asian American/Pacific Islander	28,948 (58.7)	8,321 (16.9)	12,072 (24.5)
American Indian/Alaska Native	7,089 (61.6)	1,676 (14.6)	2,741 (23.8)
Medicare and Medicaid			
Medicare Fee-for-Service only	492,453 (50.6)	191,565 (19.7)	289,794 (29.8)
Medicare FFS-Medicaid dual	215,547 (56.7)	45,781 (12.0)	119,047 (31.3)
Medicare Advantage only	316,613 (55.4)	110,226 (19.3)	144,814 (25.3)
Medicare Advantage dual	139,128 (57.1)	33,333 (13.7)	71,131 (29.2)
Urban, advantaged zip code	776,738 (52.6)	264,040 (17.9)	437,238 (29.6)
Urban, disadvantaged zip code	166,035 (58.0)	48,014 (16.8)	72,171 (25.2)
Rural, advantaged zip code	113,118 (54.2)	35,224 (16.9)	60,272 (28.9)
Rural, disadvantaged zip code	107,850 (54.9)	33,627 (17.1)	55,095 (28.0)
Chronic Conditions (CCs)			
Count of CCs (median, IQR)	5 [3,8]	6 [4,9]	7 [5,9]
ADRD/dementia	421,927 (45.2)	154,057 (16.5)	357,634 (38.3)
Ischemic Heart Disease	625,532 (47.8)	239,556 (18.3)	444,311 (33.9)

Hypertension	884,453 (50.6)	313,995 (18.0)	548,871 (31.4)
Hyperlipidemia	780,033 (49.7)	285,471 (18.2)	502,955 (32.1)
Chronic Kidney Disease	549,852 (47.0)	218,284 (18.7)	402,365 (34.4)
Depression	465,905 (47.4)	165,475 (16.8)	352,044 (35.8)
Congestive Heart Failure	481,654 (45.3)	196,384 (18.5)	385,642 (36.3)
Diabetes	460,591 (47.6)	174,372 (18.0)	332,316 (34.4)
COPD	408,958 (46.2)	162,230 (18.3)	313,146 (35.4)
Stroke/TIA	258,290 (45.8)	95,469 (16.9)	210,646 (37.3)
Cancer	230,270 (48.5)	98,407 (20.7)	146,275 (30.8)
Acute Myocardial Infarction	108,429 (44.3)	46,122 (18.9)	90,069 (36.8)
End-Stage Renal Disease	30,438 (38.0)	16,596 (20.7)	33,156 (41.4)
Hospitalizations (median, IQR)	1 [0, 3]	3 [2, 5]	4 [2, 7]
SNF days (median, IQR)	0 [0, 29]	0 [0, 27]	17 [0, 65]
≥ 100 SNF days	220,656 (61.4)	24,269 (6.8)	114,305 (31.8)

Note: Chi-squared tests for categorical variables and analyses of variance for continuous variables were all statistically significant with a p-value < 0.001. Health services utilization in the last three years was reported, except for hospice use within the last six months of life.
