# Supplementary material for: Home Health Care and Hospice Use Among Medicare Beneficiaries With and Without a Diagnosis of Dementia
Source: J Palliat Med. 2024 Jun 22;27(6):776–83. doi: 10.1089/jpm.2023.0583 (PMC11310562; doi:10.1089/jpm.2023.0583)
Supplement: Supplementary Table S2 [file jpm.2023.0583_suppl_tables2.pdf]

Table S2 Characteristics of 2019 Medicare Decedents Stratified by Dementia Diagnosis (row %)

Variable	Overall (n=2,169,422)	With Dementia (n=933,618)	No Dementia (n=1,235,804)
Hospice use (n, %)	1,151,966 (53.1)	591,272 (51.3)	560,694 (48.7)
Days in hospice (median, IQR)	2 [0, 19]	5 [0, 40]	0 [0, 10]
Mean Age at death (SD)	80.6 (10.7)	84.5 (9.4)	77.7 (10.7)
Age < 68 at death	220,320 (10.2)	44,144 (20.0)	176,176 (80.0)
Female	1,123,377 (51.8)	550,717 (49.0)	572,660 (51.0)
Male	1,046,045 (48.2)	382,901 (36.6)	663,144 (63.4)
White, non-Hispanic	1,741,205 (80.3)	762,443 (43.8)	978,762 (56.2)
Black, non-Hispanic	218,740 (10.1)	92,425 (42.3)	126,315 (57.8)
Hispanic	148,630 (6.9)	53,879 (36.3)	94,751 (63.8)
Asian American/Pacific Islander	49,341 (2.3)	20,301 (41.1)	29,040 (58.9)
American Indian/Alaska Native	11,506 (0.5)	4,570 (39.7)	6,936 (60.3)
Medicare Fee-for-Service only	973,802 (44.9)	462,167 (47.5)	511,635 (52.5)
Medicare FFS-Medicaid dual	380,375 (17.5)	251,697 (66.2)	128,678 (33.8)
Medicare Advantage only	571,653 (26.4)	125,519 (22.0)	446,134 (78.0)
Medicare Advantage dual	243,592 (11.2)	94,235 (38.7)	149,357 (61.3)
Urban, advantaged zip code	1,478,016 (68.1)	646,217 (43.7)	831,799 (56.3)
Urban, disadvantaged zip code	286,220 (13.2)	123,238 (43.1)	162,982 (56.9)
Rural, advantaged zip code	208,614 (9.6)	80,172 (38.4)	128,442 (61.6)
Rural, disadvantaged zip code	196,572 (9.1)	83,991 (42.7)	112,581 (57.3)
Count of CC (median, IQR)	6 [3, 8]	8 [6, 9]	5 [2, 7]
Ischemic Heart Disease	1,309,399 (60.4)	676,223 (51.6)	633,176 (48.4)
Hypertension	1,747,319 (80.5)	859,143 (49.2)	888,176 (50.8)
Hyperlipidemia	1,568,459 (72.3)	788,334 (50.3)	780,125 (49.7)
Chronic Kidney Disease	1,170,501 (54.0)	616,681 (52.7)	553,820 (47.3)
Depression	983,424 (45.3)	590,212 (60.0)	393,212 (40.0)

Congestive Heart Failure	1,063,680 (49.0)	566,814 (53.3)	496,866 (46.7)
Diabetes	967,279 (44.6)	482,205 (49.9)	485,074 (50.2)
COPD	884,334 (40.8)	440,962 (49.9)	443,372 (50.1)
Stroke/TIA	564,405 (26.0)	359,671 (63.7)	204,734 (36.3)
Cancer	474,948 (21.9)	217,029 (45.7)	257,919 (54.3)
Acute Myocardial Infarction	244,620 (11.3)	127,840 (52.3)	116,780 (47.7)
End-Stage Renal Disease	80,190 (3.7)	27,812 (34.7)	52,378 (65.3)
Health Services Use			
Hospitalizations (median, IQR)	2 [1, 4]	3 [1, 5]	2 [1, 4]
SNF days (median, IQR)	0 [0, 41]	24 [0, 173]	0 [0, 12]
≥ 100 SNF days	359,230 (16.6)	284,381 (79.2)	74,849 (20.8)
Home Health Care Use (n, %)	1,005,681 (46.4)	511,691 (50.9)	493,990 (49.1)
None	1,163,741 (53.6)	421,927 (36.3)	741,814 (63.7)
Began in Last Year of Life	380,905 (17.6)	154,057 (40.4)	226,848 (59.6)
Began Prior to Last Year of Life	624,776 (28.8)	357,634 (57.2)	267,142 (42.8)
Home Health Days (median, IQR)	0 [0, 57]	16 [0, 89]	0 [0, 39]

Note: *p*-value < 0.001 for all bivariate comparisons between groups with and without a diagnosis of dementia.
