# Supplementary material for: Home Health Care and Hospice Use Among Medicare Beneficiaries With and Without a Diagnosis of Dementia
Source: J Palliat Med. 2024 Jun 22;27(6):776–83. doi: 10.1089/jpm.2023.0583 (PMC11310562; doi:10.1089/jpm.2023.0583)
Supplement: Supplementary Table S3 [file jpm.2023.0583_suppl_tables3.pdf]

Table S3 Characteristics of 2019 Medicare Decedents with Dementia by Timing of Home Health Care Initiation During the Last Three Years of Life (column %), n=933,618

	None (n= 421,927)	Last Year (n= 154,057)	Prior to Last Year (n= 357,634)
Hospice use, n (%)	250,218 (59.3)	101,722 (66.0)	239,332 (66.9)
Hospice days (median, IQR)	4 [0, 37]	5 [0, 25]	7 [0,54]
Mean age at death (SD)	84.5 (9.7)	83.9 (9.0)	84.8 (9.1)
Age < 68 at death	22,034 (5.2)	6,627 (4.3)	15,483 (4.3)
Female	253,686 (60.1)	82,551 (53.6)	214,480 (60.0)
Male	168,241 (39.9)	71,506 (46.4)	143,154 (40.0)
White, non-Hispanic	343,521 (81.4)	127,029 (82.5)	291,893 (81.6)
Black, non-Hispanic	40,924 (9.7)	14,666 (9.5)	36,835 (10.3)
Hispanic	25,210 (6.0)	8,298 (5.4)	20,371 (5.7)
Asian American/Pacific Islander	9,768 (2.3)	3,408 (2.2)	7,125 (2.0)
American Indian/Alaska Native	2,504 (0.6)	656 (0.4)	1,410 (0.4)
Medicare Fee-for-Service only	177,797 (42.1)	94,279 (61.2)	190,091 (53.2)
Medicare FFS-Medicaid dual	144,912 (34.4)	23,668 (15.4)	83,117 (23.2)
Medicare Advantage only	44,723 (10.6)	26,684 (17.3)	54,112 (15.1)
Medicare Advantage dual	54,495 (12.9)	9,426 (6.1)	30,314 (8.5)
Urban, advantaged zip code	281,179 (66.6)	109,376 (71.0)	255,662 (71.5)
Urban, disadvantaged zip code	64,005 (15.2)	18,818 (12.2)	40,415 (11.3)
Rural, advantaged zip code	36,512 (8.7)	12,596 (8.2)	31,064 (8.7)
Rural, disadvantaged zip code	40,231 (9.5)	13,267 (8.6)	30,493 (8.5)
Chronic Conditions (CC)			
Count of CCs (median, IQR)	7 [5,9]	8 [6,9]	8 [6,10]
Ischemic Heart Disease	289,387 (68.6)	110,802 (71.9)	276,034 (77.2)
Hypertension	385,828 (91.4)	139,809 (90.8)	333,506 (93.3)
Hyperlipidemia	347,301 (82.3)	129,960 (84.4)	311,073 (87.0)

Chronic Kidney Disease	261,321 (61.9)	102,413 (66.5)	252,947 (70.7)
Depression	266,171 (63.1)	86,386 (56.1)	237,655 (66.5)
Congestive Heart Failure	236,559 (56.1)	91,527 (59.4)	238,728 (66.8)
Diabetes	205,746 (48.8)	76,781 (49.9)	199,678 (55.8)
COPD	182,158 (43.2)	70,434 (45.7)	188,370 (52.7)
Stroke/TIA	153,711 (36.4)	54,770 (35.6)	151,190 (42.3)
Cancer	90,489 (21.5)	39,533 (25.7)	87,007 (24.3)
Acute Myocardial Infarction	49,176 (11.7)	22,097 (14.3)	56,567 (15.8)
End-Stage Renal Disease	8,393 (2.0)	5,237 (3.4)	14,182 (4.0)
Health Services Use			
Hospitalizations (median, IQR)	2 [0,4]	3 [2,5]	4 [2,7]
SNF days (median, IQR)	37 [0, 712]	11 [0, 42]	26 [0, 95]
≥ 100 SNF days	182,694 (43.3)	15,777 (10.2)	85,910 (24.0)

Note: Chi-squared tests for categorical variables and analyses of variance for continuous variables were all statistically significant with a p-value < 0.001. Health services utilization in the last three years was reported, except for hospice use within the last six months of life.
