# Supplementary material for: Home Health Care and Hospice Use Among Medicare Beneficiaries With and Without a Diagnosis of Dementia
Source: J Palliat Med. 2024 Jun 22;27(6):776–83. doi: 10.1089/jpm.2023.0583 (PMC11310562; doi:10.1089/jpm.2023.0583)
Supplement: Supplementary Table S4 [file jpm.2023.0583_suppl_tables4.pdf]

Table S4 Characteristics of 2019 Medicare Decedents without Dementia by Timing of Home Health Care Initiation During the Last Three Years of Life (column %), n=1,235,804

	None (n=741,814)	Last Year (n=226,848)	Prior to Last Year (n=267,142)
Hospice use, n (%)	288,818 (38.9)	129,842 (57.2)	142,034 (53.2)
Hospice days (median, IQR)	0 [0, 7]	2 [0, 14]	2[0,19]
Mean age at death (SD)	77.0 (10.7)	78.3 (10.1)	79.1 (11.0)
Age < 68 at death	112,546 (15.2)	26,990 (11.9)	36,640 (13.7)
Female	320,329 (43.2)	109,099 (48.1)	143,232 (53.6)
Male	421,485 (56.8)	117,749 (51.9)	123,910 (46.4)
White, non-Hispanic	580,443 (78.3)	183,944 (81.1)	214,385 (80.3)
Black, non-Hispanic	72,642 (9.8)	23,314 (10.3)	30,359 (11.4)
Hispanic	64,974 (8.8)	13,657 (6.0)	16,120 (6.0)
Asian American/Pacific Islander	19,180 (2.6)	4,913 (2.2)	4,947 (1.9)
American Indian/Alaska Native	4,585 (0.6)	1,020 (0.5)	1,331 (0.5)
Medicare Fee-for-Service only	314,656 (42.4)	97,289 (42.9)	99,693 (37.3)
Medicare FFS-Medicaid dual	70,635 (9.5)	22,113 (9.8)	35,930 (13.5)
Medicare Advantage only	271,890 (36.7)	83,542 (36.8)	90,702 (34.0)
Medicare Advantage dual	84,633 (11.4)	23,907 (10.5)	40,817 (15.3)
Urban, advantaged zip code	495,559 (66.8)	154,664 (68.2)	181,576 (68.0)
Urban, disadvantaged zip code	102,030 (13.8)	29,196 (12.9)	31,756 (11.9)
Rural, advantaged zip code	76,606 (10.3)	22,628 (10.0)	29,208 (10.9)
Rural, disadvantaged zip code	67,619 (9.1)	20,360 (9.0)	24,602 (9.2)
Chronic Conditions (median, IQR)	4 [1,6]	5 [3, 7]	6 [3, 8]
Ischemic Heart Disease	336,145 (45.3)	128,754 (56.8)	168,277 (63.0)
Hypertension	498,625 (67.2)	174,186 (76.8)	215,365 (80.6)
Hyperlipidemia	432,732 (58.3)	155,511 (68.6)	191,882 (71.8)
Chronic Kidney Disease	288,541 (38.9)	115,871 (51.1)	149,418 (55.9)

Depression	199,734 (26.9)	79,089 (34.9)	114,389 (42.8)
Congestive Heart Failure	245,095 (33.0)	104,857 (46.2)	146,914 (55.0)
Diabetes	254,845 (34.4)	97,591 (43.0)	132,638 (49.7)
COPD	226,800 (30.6)	91,796 (40.5)	124,776 (46.7)
Stroke/TIA	104,579 (14.1)	40,699 (17.9)	59,456 (22.3)
Cancer	139,781 (18.8)	58,870 (26.0)	59,268 (22.2)
Acute Myocardial Infarction	59,253 (8.0)	24,025 (10.6)	33,502 (12.5)
End-Stage Renal Disease	22,045 (3.0)	11,359 (5.0)	18,974 (7.1)
Health Services Use			
Hospitalizations (median, IQR)	1 [0,3]	3 [2,5]	4 [2,7]
SNF days (median, IQR)	0 [0, 0]	0 [0, 20]	7 [0, 38]
≥ 100 SNF days	37,962 (5.1)	8,492 (3.7)	28,395 (10.6)

Note: Chi-squared tests for categorical variables and analyses of variance for continuous variables were all statistically significant with a p-value < 0.001. Health services utilization in the last three years was reported, except for hospice use within the last six months of life.
