# Supplementary material for: Home Health Care and Hospice Use Among Medicare Beneficiaries With and Without a Diagnosis of Dementia
Source: J Palliat Med. 2024 Jun 22;27(6):776–83. doi: 10.1089/jpm.2023.0583 (PMC11310562; doi:10.1089/jpm.2023.0583)
Supplement: Supplementary Table S5 [file jpm.2023.0583_suppl_tables5.pdf]

Table S5a. Sample Composition by Home Health Care Use During Last Three Years of Life

	Third-to-Last Year of Life	Second-to-Last Year of Life	Last Year of Life	N
No Home Health Care Use				1,163,741 (53.6%)
Started in Last Year of Life				380,905 (17.6%)
Started Prior to Last Year				213,087 (9.8%)
				130,919 (6.0%)
				130,145 (6.0%)
				60,294 (2.8%)
				55,568 (2.6%)
				34,763 (1.6%)

Table S5b. Sample Composition for Alternative (Cumulative) Indicator of Home Health Care Use

Group	Y3	Y2	Y1	
1: No HH				1,163,741 (53.6%)
2: Y1 only				380,905 (17.6%)
3: Y2 and Y1				213,087 (9.8%)
4: Y3, Y2, & Y1				130,145 (6.0%)
5: Other (Y3 & Y1, Y2 or Y3 only)				281,544 (13.0%)
