# Supplementary material for: Home Health Care and Hospice Use Among Medicare Beneficiaries With and Without a Diagnosis of Dementia
Source: J Palliat Med. 2024 Jun 22;27(6):776–83. doi: 10.1089/jpm.2023.0583 (PMC11310562; doi:10.1089/jpm.2023.0583)
Supplement: Supplementary Table S6 [file jpm.2023.0583_suppl_tables6.pdf]

Table S6. Logistic Regression Predicting Odds of Hospice Use from Alternate (Cumulative) Indicator of Home Health Use

Outcome: hospice use in the last 6 months of life (0/1) – compare with main findings in Table 3

New HH variable	With Dementia		Without Dementia	
	OR	95% CI	OR	95% CI
Group 1 (None)	Ref.		Ref.	
Group 2 (Y1)	1.34	1.32-1.35	1.92	1.90-1.94
Group 3 (Y1+Y2)	1.44	1.42-1.47	1.69	1.66-1.71
Group 4 (Y1+Y2+Y3)	1.42	1.40-1.45	1.51	1.48-1.54
Group 5 (The rest)	1.45	1.43-1.47	1.50	1.48-1.54
