# Supplementary material for: Home Health Care and Hospice Use Among Medicare Beneficiaries With and Without a Diagnosis of Dementia
Source: J Palliat Med. 2024 Jun 22;27(6):776–83. doi: 10.1089/jpm.2023.0583 (PMC11310562; doi:10.1089/jpm.2023.0583)
Supplement: Supplementary Table S7a [file jpm.2023.0583_suppl_tables7a.pdf]

Sensitivity Analyses

Table S7a. Sensitivity Analysis 1. Results of logistic regression sensitivity analysis predicting the odds of hospice use, excluding individuals who used home health services after hospice (live discharge)

Individuals who used home health care after hospice (live discharge): 671
Individuals with dementia who used home health after hospice: 381
Individuals without dementia who used home health after hospice: 290

Original Model	All Decedents		With Dementia		Without Dementia	
	n= 2,169,422		n=933,618		n=1,235,804	
	OR	95% CI	OR	95% CI	OR	95% CI
Home Health Use (Ref = none)						
Started prior to last year	1.57	1.56-1.58	1.44	1.43-1.46	1.56	1.54-1.58
Started during last year of life	1.75	1.74-1.77	1.34	1.32-1.35	1.92	1.90-1.94
Sensitivity Analyses	Decedents excluding those who used home health after hospice		Decedents with dementia excluding those who used home health after hospice		Decedents without dementia excluding those who used home health after hospice	
	N=2,168,751		N=933,237		N=1,235,514	
Home Health Use (Ref = none)						
Started prior to last year	1.57	1.56-1.58	1.44	1.43-1.46	1.56	1.54-1.57
Started during last year of life	1.75	1.74-1.76	1.33	1.32-1.35	1.92	1.90-1.94
