# Supplementary material for: Home Health Care and Hospice Use Among Medicare Beneficiaries With and Without a Diagnosis of Dementia
Source: J Palliat Med. 2024 Jun 22;27(6):776–83. doi: 10.1089/jpm.2023.0583 (PMC11310562; doi:10.1089/jpm.2023.0583)
Supplement: Supplementary Table S7b [file jpm.2023.0583_suppl_tables7b.pdf]

Table S7b. Sensitivity Analysis 2. Results of logistic regressions predicting the odds ratios of hospice use on a sample excluding individuals who died in a nursing home

Individuals who died in a nursing home: 121,669
Individuals with dementia who died in a nursing home: 83,354
Individuals without dementia who died in a nursing home: 38,315

Original Model	All Decedents		With Dementia		Without Dementia	
	n= 2,169,422		n=933,618		n=1,235,804	
	OR	95% CI	OR	95% CI	OR	95% CI
Home Health Use (Ref = none)						
Started before last year	1.57	1.56-1.58	1.44	1.43-1.46	1.56	1.54-1.58
Started in last year of life	1.75	1.74-1.77	1.34	1.32-1.35	1.92	1.90-1.94
Sensitivity Analyses	Decedents excluding those who died in nursing home		Decedents with dementia excluding those who died in nursing home		Decedents without dementia excluding those who died in nursing home	
	N=2,047,753		N=850,264		N=1,197,489	
Home Health Use (Ref = none)						
Started before last year	1.57	1.55-1.58	1.41	1.40-1.43	1.58	1.56-1.59
Started in last year of life	1.77	1.76-1.79	1.30	1.28-1.32	1.95	1.93-1.97
