# Supplementary material for: Home Health Care and Hospice Use Among Medicare Beneficiaries With and Without a Diagnosis of Dementia
Source: J Palliat Med. 2024 Jun 22;27(6):776–83. doi: 10.1089/jpm.2023.0583 (PMC11310562; doi:10.1089/jpm.2023.0583)
Supplement: Supplementary Table S8a [file jpm.2023.0583_suppl_tables8a.pdf]

Table S8a. Logistic Regression for Hospice Use with Interaction (Home Health Use x Dementia)

	All Decedents (n=2,169,422)	
	Adjusted Odds Ratio	95% CI
Home Health Use (Ref = none)		
Started prior to last year	1.78	1.76-1.80
Started in last year of life	2.08	2.06-2.10
Dementia Diagnosis	2.14	2.12-2.16
Home Heath ## Dementia		
Started prior to the last year # Dementia	0.75	0.74-0.76
Started in last year of life # Dementia	0.62	0.61-0.63
