# Supplementary material for: Home Health Care and Hospice Use Among Medicare Beneficiaries With and Without a Diagnosis of Dementia
Source: J Palliat Med. 2024 Jun 22;27(6):776–83. doi: 10.1089/jpm.2023.0583 (PMC11310562; doi:10.1089/jpm.2023.0583)
Supplement: Supplementary Table S8b [file jpm.2023.0583_suppl_tables8b.pdf]

Table S8b. Predicted Probability of Hospice Use by Home Health Use and a Dementia Diagnosis

| | Predicted probability of hospice use | 95% CI |
|---|---|---|
| No HH | 0.48 | 0.48-0.48 |
| Started HH prior to Last Year | 0.58 | 0.58-0.59 |
| Started HH in Last Year | 0.60 | 0.60-0.60 |
| | | |
| Without Dementia | 0.48 | 0.47-0.48 |
| With Dementia | 0.61 | 0.61-0.61 |
| | | |
| No HH # Dementia | 0.58 | 0.58-0.58 |
| HH Started prior to last year # Dementia | 0.64 | 0.64-0.65 |
| HH Started in last year # Dementia | 0.64 | 0.64-0.64 |
| No HH # Without Dementia | 0.41 | 0.41-0.41 |
| HH Started prior to last year # Without Dementia | 0.54 | 0.54-0.54 |
| HH Started in last year # Without Dementia | 0.57 | 0.57-0.58 |
