# Supplementary material for: Home Health Care and Hospice Use Among Medicare Beneficiaries With and Without a Diagnosis of Dementia
Source: J Palliat Med. 2024 Jun 22;27(6):776–83. doi: 10.1089/jpm.2023.0583 (PMC11310562; doi:10.1089/jpm.2023.0583)
Supplement: Supplementary Table S9 [file jpm.2023.0583_suppl_tables9-s12.pdf]

Table S9. Multinomial Logistic Regression of Hospice Use for Various Lengths of Time for Decedents with Dementia (1-2, 3-179, 180+ hospice days)

	1-2 Hospice Days Vs. No Hospice		3-179 Hospice Days vs. No Hospice		180+ Hospice Days vs. No Hospice	
	RRR	95% CI	RRR	95% CI	RRR	95% CI
Home Health Use (Ref= none)						
Started prior to last year	1.11	1.09-1.14	1.48	1.47-1.50	1.68	1.65-1.71
Started in last year of life	1.31	1.28-1.35	1.54	1.52-1.56	0.32	0.31-0.33
Race/Ethnicity (Ref = white)						
Black	0.58	0.56-0.60	0.73	0.72-0.74	0.64	0.62-0.66
Hispanic	0.81	0.77-0.84	0.85	0.83-0.87	0.76	0.73-0.79
AAPI	0.71	0.66-0.77	0.66	0.63-0.68	0.53	0.50-0.57
AIAN	0.70	0.61-0.80	0.72	0.67-0.76	0.60	0.53-0.69
Age at death (centered)	1.02	1.01-1.02	1.02	1.02-1.02	1.03	1.03-1.04
Age < 68 at death	0.85	0.81-0.90	0.81	0.79- 0.84	0.96	0.91-1.02
Female (Ref = male)	0.98	0.96-1.00	1.16	1.14-1.17	1.40	1.37-1.42
Medicare Fee-for-Service only	Ref.	Ref.	Ref.	Ref.	Ref.	Ref.
Medicare FFS-Medicaid dual	0.89	0.86-0.91	0.93	0.92-0.94	1.22	1.19-1.24
Medicare Advantage only	1.09	1.06-1.13	1.32	1.30-1.34	1.42	1.38-1.46
Medicare Advantage dual	0.83	0.80-0.87	1.00	0.98-1.01	1.19	1.15-1.22
Urban, advantaged zip code	Ref.	Ref.	Ref.	Ref.	Ref.	Ref.
Urban, disadvantaged zip code	0.79	0.77-0.81	0.77	0.76-0.78	0.69	0.67-0.71
Rural, advantaged zip code	0.87	0.85-0.90	0.85	0.84-0.87	0.77	0.75-0.80
Rural, disadvantaged zip code	0.81	0.78-0.84	0.79	0.78-0.81	0.71	0.69-0.74
Chronic Conditions						
Ischemic Heart Disease	0.97	0.95-0.99	0.93	0.91-0.94	0.91	0.90-0.93
Hypertension	0.89	0.85-0.93	0.86	0.84-0.88	0.93	0.90-0.96
Hyperlipidemia	1.00	0.97-1.03	1.05	1.03-1.06	0.95	0.92-0.97

Chronic Kidney Disease	1.07	1.05-1.10	0.90	0.89-0.91	0.76	0.74-0.77
Depression	0.97	0.95-0.99	1.17	1.16-1.19	1.54	1.51-1.56
Congestive Heart Failure	1.02	0.99-1.04	0.82	0.81-0.83	0.87	0.85-0.89
Diabetes	0.94	0.93-0.96	0.92	0.91-0.93	0.91	0.89-0.92
COPD	0.96	0.94-0.99	0.84	0.84-0.85	0.92	0.90-0.93
Stroke/TIA	1.06	1.04-1.08	1.05	1.04-1.06	1.12	1.10-1.14
Cancer	1.10	1.03-1.17	1.14	1.10-1.18	1.04	0.97-1.10
AMI	0.90	0.87-0.92	0.85	0.84-0.86	0.79	0.77-0.82
End-Stage Renal Disease	0.84	0.80-0.88	0.62	0.61-0.64	0.12	0.10-0.14
Health Services Used						
≥ 100 SNF days	1.01	0.98-1.03	0.90	0.89- 0.92	0.56	0.55-0.58
Hospitalizations	1.03	1.02-1.03	0.99	0.99-0.99	0.80	0.80-0.80

Note: RRR= relative risk ratio. 95% CI=95% Confidence Interval. Models were adjusted for state and cancer subtype.

Table S10. Multinomial Logistic Regression of Hospice Use for Various Lengths of Time for Decedents with Dementia (1-7, 8-179, 180+ hospice days)

	1-7 Hospice Days vs. No Hospice		8-179 Hospice Days vs. No Hospice		180+ Hospice Days vs. No Hospice	
	RRR	95% CI	RRR	95% CI	RRR	95% CI
Home Health Use (Ref= none)						
Started prior to last year	1.19	1.17-1.20	1.598	1.58-1.62	1.69	1.66-1.72
Started in last year of life	1.40	1.37-1.42	1.586	1.56-1.61	0.32	0.31-0.33
Race/Ethnicity (Ref = white)						
Black	0.61	0.60-0.63	0.77	0.76-0.79	0.64	0.62-0.66
Hispanic	0.79	0.77-0.81	0.85	0.83-0.87	0.76	0.73-0.79
AAPI	0.719	0.69-0.75	0.63	0.61-0.65	0.53	0.50-0.57
AIAN	0.70	0.64-0.76	0.72	0.67- 0.78	0.60	0.53-0.69
Age at death (centered)	1.02	1.01-1.02	1.02	1.02-1.02	1.03	1.03-1.04
Age < 68 at death	0.83	0.80-0.86	0.81	0.79-0.84	0.96	0.91-1.02

Female (Ref = male)	1.03	1.02-1.05	1.20	1.18-1.21	1.40	1.37-1.42
Medicare Fee-for-Service only	Ref.	Ref.	Ref.	Ref.	Ref.	Ref.
Medicare FFS-Medicaid dual	0.89	0.87-0.90	0.95	0.94-0.96	1.22	1.19-1.25
Medicare Advantage only	1.17	1.14-1.19	1.36	1.34-1.39	1.42	1.39-1.46
Medicare Advantage dual	0.89	0.87-0.91	1.03	1.01-1.05	1.19	1.16-1.23
Urban, advantaged zip code	Ref.	Ref.	Ref.	Ref.	Ref.	Ref.
Urban, disadvantaged zip code	0.79	0.77-0.80	0.76	0.75-0.78	0.69	0.67-0.71
Rural, advantaged zip code	0.87	0.85-0.89	0.85	0.83-0.86	0.77	0.75-0.80
Rural, disadvantaged zip code	0.81	0.79-0.83	0.79	0.77-0.80	0.71	0.69-0.74
Chronic Conditions						
Ischemic Heart Disease	0.96	0.94-0.97	0.92	0.91-0.93	0.91	0.90-0.93
Hypertension	0.85	0.83-0.87	0.86	0.85 0.88	0.93	0.90-0.96
Hyperlipidemia	1.02	1.00-1.04	1.05	1.04-1.07	0.95	0.93-0.97
Chronic Kidney Disease	1.02	1.00-1.03	0.87	0.85-0.88	0.76	0.74-0.77
Depression	1.02	1.01-1.04	1.23	1.21-1.24	1.54	1.51-1.57
Congestive Heart Failure	0.91	0.90-0.93	0.80	0.79-0.81	0.87	0.85-0.89
Diabetes	0.93	0.91-0.94	0.92	0.91-0.93	0.91	0.89-0.92
COPD	0.89	0.88-0.90	0.84	0.83-0.85	0.92	0.90-0.93
Stroke/TIA	1.08	1.08-1.10	1.03	1.02-1.04	1.12	1.10-1.14
Cancer	1.12	1.08-1.17	1.14	1.10-1.18	1.04	0.97-1.10
AMI	0.88	0.87-0.90	0.84	0.82-0.85	0.79	0.77-0.82
End-Stage Renal Disease	0.97	0.94-0.99	0.44	0.43-0.46	0.12	0.10-0.14
Health Services Used						
≥ 100 SNF days	0.95	0.94-0.97	0.90	0.89- 0.91	0.56	0.55-0.58
Hospitalizations	1.02	1.02-1.02	0.98	0.98-0.98	0.80	0.80-0.80

Table S11. Multinomial Logistic Regression of Hospice Use for Various Lengths of Time for Decedents without Dementia (1-2, 3-179, 180+ hospice days)

	1-2 Hospice Days Vs. No Hospice		3-179 Hospice Days vs. No Hospice		180+ Hospice Days vs. No Hospice	
	RRR	95% CI	RRR	95% CI	RRR	95% CI
Home Health Use (Ref = none)						
Started prior to last year	1.13	1.11-1.16	1.57	1.55-1.58	2.81	2.74-2.88
Started in last year of life	1.62	1.59-1.65	2.08	2.05-2.10	0.67	0.64-0.70
Race/Ethnicity (Ref = white)						
Black	0.57	0.56- 0.59	0.68	0.67-0.69	0.56	0.54-0.59
Hispanic	0.79	0.76-0.82	0.82	0.81-0.84	0.79	0.75-0.83
AAPI	0.75	0.71-0.80	0.73	0.71-0.75	0.60	0.55-0.65
AIAN	0.78	0.70- 0.88	0.77	0.73-0.82	0.67	0.57-0.79
Age at death (centered)	1.03	1.03-1.03	1.04	1.04-1.04	1.07	1.07-1.07
Age < 68 at death	0.88	0.85-0.91	0.96	0.94-0.97	1.55	1.47-1.62
Female (Ref = Male)	1.11	1.09-1.13	1.20	1.19-1.21	1.35	1.32-1.39
Medicare Fee-for-Service only	Ref.	Ref.	Ref.	Ref.	Ref.	Ref.
Medicare FFS-Medicaid dual	0.94	0.92-0.97	1.04	1.03-1.06	1.63	1.57-1.70
Medicare Advantage only	1.47	1.44-1.50	1.38	1.37-1.40	1.48	1.44-1.53
Medicare Advantage dual	1.26	1.22-1.29	1.30	1.28-1.32	1.66	1.59-1.72
Urban, advantaged zip code	Ref.	Ref.	Ref.	Ref.	Ref.	Ref.
Urban, disadvantaged zip code	0.87	0.85-0.89	0.92	0.91-0.93	0.87	0.84-0.90
Rural, advantaged zip code	0.92	0.89-0.94	0.91	0.89-0.92	0.89	0.86-0.93
Rural, disadvantaged zip code	0.85	0.83-0.88	0.90	0.88-0.91	0.84	0.81-0.88
Chronic Conditions						
Ischemic Heart Disease	0.96	0.94-0.98	0.92	0.91-0.93	0.99	0.96-1.02
Hypertension	0.97	0.94-0.99	0.95	0.93-0.96	0.94	0.91-0.97
Hyperlipidemia	0.96	0.94-0.98	1.00	0.99-1.01	0.81	0.78-0.83

Chronic Kidney Disease	1.21	1.18-1.23	1.01	1.00-1.02	0.86	0.83-0.88
Depression	0.96	0.94-0.98	1.17	1.16-1.18	1.51	1.47-1.55
Congestive Heart Failure	1.14	1.12-1.16	0.88	0.87-0.89	1.15	1.12-1.18
Diabetes	0.88	0.86-0.89	0.87	0.86-0.88	0.86	0.84-0 .89
COPD	1.03	1.01-1.05	0.95	0.94-0.96	1.46	1.43- 1.50
Stroke/TIA	1.17	1.15-1.19	1.03	1.02-1.04	1.05	1.02-1.08
Cancer	1.39	1.32-1.46	1.69	1.64-1.74	1.52	1.41-1.63
AMI	0.80	0.78-0.82	0.78	0.77-0.79	0.82	0.79-0.85
End-Stage Renal Disease	0.79	0.76-0.82	0.55	0.54-0.57	0.14	0.12-0.17
Health Services Used						
≥ 100 SNF days	0.98	0.95-1.01	0.97	0.96-0.99	0.74	0.71-0.78
Hospitalizations	1.08	1.08-1.09	1.05	1.05-1.05	0.83	0.82-0.83

Note: RRR= relative risk ratio. 95% CI=95% Confidence Interval. Models were adjusted for state and cancer subtype.

Table S12. Multinomial Logistic Regression of Hospice Use for Various Lengths of Time for Decedents without Dementia (1-7, 8-179, 180+ hospice days)

	1-7 Hospice Days Vs. No Hospice		8-179 Hospice Days vs. No Hospice		180+ Hospice Days vs. No Hospice	
	RRR	95% CI	RRR	95% CI	RRR	95% CI
Home Health Use (Ref = none)						
Started prior to last year	1.225	1.21-1.24	1.72	1.70-1.74	2.82	2.75-2.89
Started in last year of life	1.828	1.80-1.85	2.14	2.11-2.16	0.67	0.64-0.70
Race/Ethnicity (Ref = white)						
Black	0.60	0.59-0.61	0.71	0.70-0.72	0.56	0.54-0.59
Hispanic	0.78	0.76-0.80	0.85	0.83-0.86	0.79	0.75-0.83
AAPI	0.71	0.68-0.74	0.74	0.72-0.77	0.60	0.55-0.65
AIAN	0.77	0.72- 0.83	0.77	0.72-0.82	0.67	0.57-0.79
Age at death (centered)	1.03	1.03-1.03	1.04	1.04-1.04	1.07	1.07-1.07
Age < 68 at death	0.89	0.87-0.91	0.99	0.97-1.00	1.55	1.47-1.62

Female (Ref = Male)	1.13	1.11-1.14	1.23	1.21-1.24	1.36	1.33-1.39
Medicare Fee-for-Service only	Ref.	Ref.	Ref.	Ref.	Ref.	Ref.
Medicare FFS-Medicaid dual	0.94	0.92-0.96	1.09	1.07-1.11	1.64	1.57-1.70
Medicare Advantage only	1.44	1.42-1.46	1.36	1.35-1.38	1.48	1.44-1.53
Medicare Advantage dual	1.26	1.23-1.28	1.32	1.30-1.34	1.66	1.60-1.72
Urban, advantaged zip code	Ref.	Ref.	Ref.	Ref.	Ref.	Ref.
Urban, disadvantaged zip code	0.90	0.88-0.91	0.92	0.91-0.94	0.87	0.84-0.90
Rural, advantaged zip code	0.92	0.90-0.93	0.90	0.89-0.92	0.89	0.86-0.93
Rural, disadvantaged zip code	0.87	0.85-0.89	0.90	0.89-0.92	0.84	0.81-0.88
Chronic Conditions						
Ischemic Heart Disease	0.94	0.93- 0.95	0.92	0.91-0.93	0.99	0.96-1.02
Hypertension	0.96	0.94- 0.98	0.94	0.93-0.95	0.94	0.91-0.97
Hyperlipidemia	0.99	0.97-1.00	1.00	0.99-1.01	0.81	0.78-0.83
Chronic Kidney Disease	1.16	1.14-1.17	0.96	0.95-0.97	0.86	0.83-0.88
Depression	1.03	1.02-1.05	1.22	1.21-1.23	1.51	1.48-1.55
Congestive Heart Failure	0.99	0.98-1.01	0.86	0.85-0.87	1.15	1.12-1.18
Diabetes	0.88	0.87-0.89	0.87	0.86-0.88	0.86	0.84-0.89
COPD	0.96	0.95-0.97	0.96	0.95-0.97	1.46	1.43- 1.50
Stroke/TIA	1.15	1.14-1.17	0.98	0.97-0.99	1.05	1.02-1.08
Cancer	1.435	1.38-1.49	1.80	1.75-1.86	1.52	1.41- 1.63
AMI	0.78	0.77-0.80	0.78	0.77-0.80	0.82	0.79- 0.85
End-Stage Renal Disease	0.83	0.81-0.86	0.40	0.38-0.41	0.14	0.12-0.16
Health Services Used						
≥ 100 SNF days	0.99	0.97-1.01	0.97	0.94-0.98	0.74	0.70-0.78
Hospitalizations	1.08	1.08-1.08	1.03	1.03-1.03	0.83	0.82-0.83
