# Supplementary material for: Home Health Care and Hospice Use Among Medicare Beneficiaries With and Without a Diagnosis of Dementia
Source: J Palliat Med. 2024 Jun 22;27(6):776–83. doi: 10.1089/jpm.2023.0583 (PMC11310562; doi:10.1089/jpm.2023.0583)
Supplement: Supplementary file 13 [file jpm.2023.0583_suppl_texts1.pdf]

Text S1. Methodology of Calculating Home Health Care Days

The main independent variable was a three-category indicator of the timing of home health use: never used home health care in the last three years of life, began using home health care in the last year, and used prior to the last year during the last three years. To calculate home health days, we retained assessment dates and assessment reasons from the OASIS file for our sample during the study period of 2016 to 2019. After sorting the assessment dates in reverse chronological order and creating a lag variable that preserved the date for the next assessment date alongside the prior assessment date, we calculated the number of home health days by subtracting the next assessment dates from the prior assessment dates. Additional rules were employed to address specific cases identified in the data. For assessments with reasons indicating the start of care or resumption of care, if the calculated days exceeded 60 days, we assigned a value of 60 days. In the case of reassessments for follow-up, if the calculated days exceeded 65 days, we capped it at a value of 65 days. We applied this rule because it would be unreasonable to assume that patients received home health care for that long period of time, and the unexpectedly long calculated home health days may be attributed to a gap in the continuity of home health care between those assessments.
