# Supplementary material for: Home Health Care and Hospice Use Among Medicare Beneficiaries With and Without a Diagnosis of Dementia
Source: J Palliat Med. 2024 Jun 22;27(6):776–83. doi: 10.1089/jpm.2023.0583 (PMC11310562; doi:10.1089/jpm.2023.0583)
Supplement: Supplementary file 14 [file jpm.2023.0583_suppl_texts2.pdf]

Text S2. Methodology of Identifying Diagnosis of Alzheimer's Disease and Other Dementias

Alzheimer's disease and related dementia (ADRD) were identified using the International Classification of Diseases, Tenth Revision (ICD-10) and Ninth Revision (ICD-9) diagnosis codes (331.0, 331.11, 331.19, 331.7, 331.2, 290.0, 290.11, 290.13, 290.20, 290.21, 290.3, 290.40, 290.41, 290.42, 290.43, 294.0, 294.10, 290.11, 294.20, 294.21, 294.8, 797, G30.0, G30.1, G30.8, G30.9, F01.50, F01.51, F02.80, F02.81, F03.90, F03.91, F04, G13.8, F05, F06.1, F06.8, G30.0, G30.1, G30.8, G30.9, G31.1, G31.2, G31.01, G31.09, G94, R41.81, and R54). We augmented the Master Beneficiary Summary File Chronic Conditions segment by additionally leveraging available diagnostic information from MedPAR, OASIS, and MDS to identify if patients were diagnosed with dementia.
